# Chemical Elements, Flavor Chemicals, and Nicotine in Unused and Used Electronic Cigarettes Aged 5–10 Years and Effects of pH

**DOI:** 10.3390/ijerph192416931

**Published:** 2022-12-16

**Authors:** Monique Williams, Wentai Luo, Kevin McWhirter, Omeka Ikegbu, Prue Talbot

**Affiliations:** 1 Department of Molecular, Cell and Systems Biology, University of California, Riverside, CA 92521, USA; 2 Department of Chemistry, Portland State University, Portland, OR 97207, USA; 3 Department of Civil & Environmental Engineering, Portland State University, Portland, OR 97207, USA

**Keywords:** metals, elements, electronic cigarettes, ENDS, flavor chemicals, nicotine, pH, acids, aging, leaching, environmental waste

## Abstract

The concentrations of elements/metals, nicotine, flavor chemicals and acids were compared in the e-liquids of unused and used first-generation electronic cigarettes (ECs) that were stored for 5–10 years. Metal analysis was performed using inductively coupled plasma optical emission spectroscopy; nicotine and flavor chemical analyses were performed using gas chromatography/mass spectroscopy. Of the 22 elements analyzed, 10 (aluminum, chromium, copper, iron, lead, nickel, selenium, silicon, tin, zinc) were often found in the e-liquids. Five elements had the highest average concentrations: copper (1161.6 mg/L), zinc (295.8 mg/L), tin (287.6 mg/L), nickel (71.1 mg/L), and lead (50.3 mg/L). Nicotine concentrations were always lower than label concentrations indicated. Of the 181 flavor chemicals analyzed, 11 were detected in at least one sample, with hydroxyacetone being present in all samples. In used products, some flavor chemicals appeared to be by-products of heating. E-liquids with the highest concentrations of acids and the lowest pH levels also had the highest concentrations of elements/metals. Metal concentrations in e-liquids increased after use in some products, and some metal concentrations, such as nickel, were high enough to be a health concern. Leachates from discarded ECs could contribute toxic metals/chemicals to the environment, supporting the need for better regulation of atomizer design, composition, and disposal.

## 1. Introduction

Electronic cigarettes (ECs) contain atomizing units that are comprised of elements/metals. Some atomizing unit components are preserved in all generations of ECs [1,2,3,4], while only fourth-generation pod-style ECs contain connector plates/pins and magnets [5]. Atomizer components usually include wires (copper, silver), a filament (nickel, chromium), wick (silicon), wire joints (which can be brass clamps—copper, zinc, solder (tin, lead), or braised wires) [1,2,4,6]. Some atomizer elements, such as chromium, lead, and nickel, are carcinogens and respiratory toxicants [7,8,9,10]. Conversely, other atomizer elements, such as calcium, potassium, and magnesium, are less likely to cause harm [2,4,11].

Refill fluids also contain elements/metals [11,12,13,14], some of which (selenium, aluminum, tin, arsenic, chromium, lead, nickel, zinc, copper, manganese) are present before use and are known to be harmful [11,12,13,14,15,16,17]. Selenium, which is an impurity of propylene glycol and glycerin, can cause cytotoxicity to bronchial epithelial cells [11] and is on the Federal Drug Administration’s (FDA) Harmful and Potentially Harmful list and the Agency for Toxic Substances and Disease Research’s (ATSDR) Priority List of Hazardous Substances list [7,15]. Concentrations of some elements (copper, manganese, zinc, nickel) are higher in e-liquids after use, presumably because they are released from the atomizing units during heating [11,12,18,19,20]. In addition to elements/metals, the fluids also contain nicotine and numerous flavor chemicals [21,22,23,24,25,26]. EC refill fluids come in a variety of nicotine concentrations and flavors. The most popular refill fluids contain numerous flavor chemicals, such as cinnamaldehyde, ethyl maltol, vanillin, and benzyl alcohol, which are often used at high concentration that are cytotoxic to respiratory epithelium [22,24,25,27,28,29,30,31].

It is not known how use, aging, and storage affect the concentrations of elements/metals, nicotine, and flavor chemicals in e-liquids. The purpose of this study was to determine element/metal concentrations in the fluid of first-generation ECs that have aged for 5–10 years, compare element/metal concentrations in unused and used fluid of 10 different EC brands, and identify and quantify the concentrations of nicotine and flavor chemicals in aged unused and used first-generation e-liquids. The effects of e-liquid pH on element/metal concentrations are also examined.

## 2. Materials and Methods

### 2.1. Electronic Cigarette Selection and Experimental Design

To compare the effects of aging on metal, nicotine, and flavor chemical concentrations, fluid from 10 brands of first-generation ECs, which had been stored at room temperature for 5–10 years, were selected. Eight brands of cartomizer-style ECs (BluCig, Greensmoke, Mark Ten, NJOY NPRO, SafeCig, South Beach Smoke, V2 Cigs, and Vuse), and two brands of disposable-style ECs (BluCig and Vype) were used (Table S1). MarkTen, Vuse, and Vype products were aged at least 5 years, while all other brands had aged about 10 years. All EC brands were tobacco-flavored, except for BluCig which also included menthol flavor (BluCig Menthol). Cartomizers and disposable inventory were divided into three categories: unused (0 puffs), gently used (10–60 puffs), and heavily used (61–450 puffs). Continuous puffing was done on a smoking machine as described previously [1,2,6,32]. There were three exceptions that did not have all three categories: BluCig Menthol only had unused fluid, SafeCig and Vype only had unused and gently used fluid, and MarkTen and Vuse only had gently and heavily used fluid. For three brands (BluCig, NJOY NPRO, SafeCig), metal analysis was repeated with unused samples to validate concentration data.

### 2.2. Fluid Isolation and Metal Analysis Sample Preparation

EC cartomizers/disposables from 10 brands and each category (unused, gently used, heavily used) were dissected, and fluid was isolated from the ECs as described previously [3,11]. The fluid samples were prepared by dissolving 500 µL of e-liquid into 9.5 mL of 98% deionized water and 2% nitric acid (Table S1) [11,12,13]. All samples were prepared and stored in nitric acid-washed and sealed 15 mL conical vials, and then were immediately analyzed after preparation. Twenty-two elements were screened in the fluids using inductively coupled plasma optical emission spectroscopy (ICP-OES), as described previously [2,3,11]. In addition, a standard curve was prepared (0.000 to 10,000 mg/L) for each of the 22 elements. A 2% nitric acid blank was analyzed, and concentrations in the blank were subtracted from all test samples. For every brand and category, the samples were analyzed in triplicate. A full description of ICP-OES running conditions is described in the Supplemental Materials section.

### 2.3. Flavor Chemicals and Nicotine in Unused and Used E-Liquid

All aged e-liquid were prepared for flavor chemical analysis, as described in detail previously [24,25]. All samples were prepared at a 1:20 dilution by dissolving 50 µL of e-liquid into 950 µL of isopropyl alcohol (Table S1). Samples were prepared and stored in amber GC vials. A total of 181 flavor chemicals and nicotine were screened in the aged fluids using gas chromatography/mass spectrometry (GC-MS), which was performed with an Agilent 5975 C GC/MS system (Santa Clara, CA, USA). An isopropyl alcohol blank was also analyzed. Additional running conditions and instrument information for the GC-MS are given in the Supplemental Material.

### 2.4. pH Measurements in Aged Unused E-Liquids

All aged unused e-liquids were prepared for pH measurements. All samples were diluted 1:20 by dissolving 50 µL of e-liquid in 950 µL of deionized water (Table S1). A calibrated pH meter was used to measure pH in each fluid.

### 2.5. Acid Identification and Quantification in Aged Unused E-Liquid

All aged unused and used e-liquids were prepared to identify acids. Authentic reference material for each target organic acid was dissolved in a 50%/50% mixture of HPLC grade-water and methanol to produce a stock solution. These stock solutions were diluted in mobile phase A (see below) to produce a multipoint calibration standard ranging in concentration from ~20 ng/µL to ~500 ng/µL for each target acid. Samples were prepared for analysis by diluting 20 µL of refill fluid with mobile phase A to 1000 µL. The diluted refill fluid samples were shaken by hand until mixed, and then analyzed immediately.

Analyses were completed using an Agilent Technologies (Santa Clara, CA, USA) Infinity 1260 HPLC with a UV-VIS detector. The wavelengths 210 nm and 230 nm (bandwidth 4 nm) were used for detection, with 360 nm (bandwidth 80 nm) as the reference wavelength. An Agilent InfinityLab Poroshell 120 SB-AQ column (3.0 × 150 mm and 2.7-micron particle size) was used for separation. The analytical column was protected by a 3.0 × 5 mm guard column with the same particle size. The column chamber was kept at 35 °C for the duration of the run. The injection volume was 2 µL. The flow rate was 0.500 mL/min. Mobile phase A was prepared with a pH 2 phosphate buffer in HPLC-grade water with 1% HPLC-grade acetonitrile, and mobile phase B was 100% HPLC-grade acetonitrile. The mobile phase gradient used was as follows: 100% A from start until 4.5 min, then grade to 40% A at 11.5 min until 16 min, and then 100% A at 16.1 min until 20 min.

## 3. Results

### 3.1. Frequency of Individual Elements Present in E-Liquids

**Figure 1:**
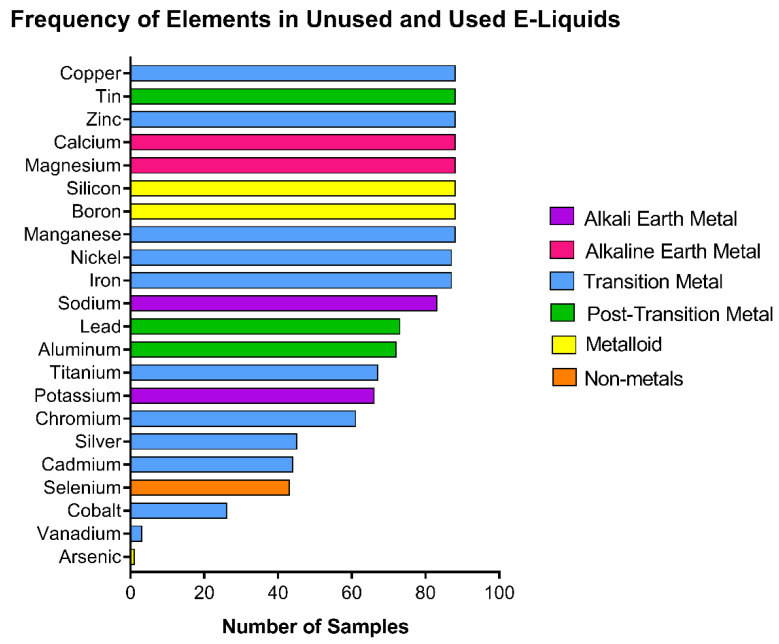
Frequency of elements in unused, gently used, and heavily used e-liquids. Hierarchy of 22 elements screened in 89 samples from 10 brands of first-generation e-liquids. Color coded by periodic table group.

Twenty-two elements were screened in the fluids of 10 unused and used EC brands that had been stored at room temperature for 5–10 years (Figure 1). A total of 89 samples of first-generation EC cartomizers/disposables were evaluated. Twenty elements were detected at least once in the samples. The most frequently found elements were calcium, copper, magnesium, manganese, silicon, boron, tin, zinc, iron, nickel, and sodium, all of which were each detected in over 80 samples (Figure 1). In contrast, vanadium was measured in three samples, and arsenic was found in one sample. Various classes of elements were identified. Non-metals included selenium; metalloids included boron, silicon, and arsenic; post-transition metals included aluminum, tin, and lead; transition metals included copper, manganese, zinc, iron, nickel, chromium, silver, cadmium, cobalt, and vanadium; alkaline earth metals included calcium and magnesium; alkali metals included sodium and potassium.

### 3.2. Total Concentrations of Elements/Metals in Aged Unused and Used E-Liquids

The total concentration of the 22 elements varied among brands (Table 1 and Table S2). The highest total concentrations were found in unused NJOY NPRO (2214.86 mg/L), unused SafeCig (1661.17 mg/L), gently used Greensmoke (1478.32 mg/L), and heavily used Greensmoke (1292.95 mg/L). Conversely, the lowest total concentrations were found in unused V2 Cig (11.96 mg/L), unused South Beach (10.07 mg/L), heavily used Vuse (6.91 mg/L), and gently used Vuse (5.65 mg/L) (Table 1). Potentially toxic elements that were detected at average concentrations >1 mg/L included copper, iron, lead, nickel, silicon, tin, and zinc (Table 1).

### 3.3. Concentrations of Individual Elements/Metals in Aged Unused and Used E-Liquids

The concentration of the 22 elements was compared and varied among the 10 brands (Table 1 and Tables S2–S4). Ranges for each element are summarized in Supplemental Table S5. The seven highest elements found in used and unused Greensmoke cartomizer fluid were aluminum, boron, chromium, iron, manganese, nickel and tin (Tables S6 and S7). Conversely, copper, lead, silver, sodium, and zinc were the highest in NJOY NPRO products (Tables S6 and S7). The highest concentrations of the remaining 10 elements were expressed as follows: in BluCig (magnesium, titanium), BluCig Disposable (potassium, vanadium), SafeCig (cadmium, cobalt), V2 Cigs (calcium, silicon), and Vype (arsenic, selenium) (Tables S6 and S7).

The concentrations of the elements were next examined independently of brands (Table S7). Copper had the highest concentration (1749.64 mg/L), followed by sodium (979.46 mg/L), zinc (528.67 mg/L), and tin (420.41 mg/L). Relatively high concentrations of nickel (102.49 mg/L) and lead (93.38 mg/L), two of the most toxic elements, were present in both aged unused and used fluids. Silicon, iron, aluminum, potassium, boron, and magnesium were detected at concentrations of less than 12.64 mg/L (Table S7). The nine remaining elements (manganese, selenium, chromium, silver, cobalt, titanium, cadmium, arsenic, vanadium) were all present at concentrations <0.79 mg/L (Table S7). Graphical data for each brand and each element are shown in Supplemental Figures S1–S22.

Of the 22 elements detected in the aged unused and used e-liquids, 16 have been previously identified in the atomizer components of first-generation ECs (Table S8) [4].

### 3.4. Comparison of Metal Concentrations in Unused and Used E-Liquids

**Figure 2:**
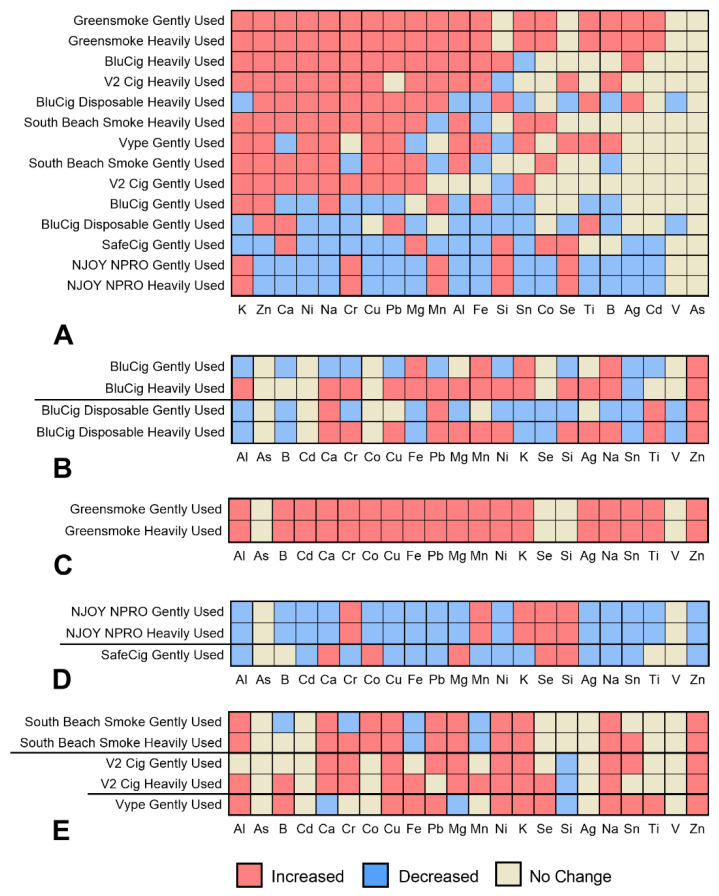
Heat map showing element concentrations in gently or heavily used ECs relative to the unused products. (**A**) Hierarchy of 10 brands (top to bottom) and 22 elements (left to right) where the concentration of gently or heavily used fluids increased in comparison to that of the unused. (**B**) Examples in which concentration increased in the heavily used compared to the gently used and unused fluid. (**C**) Brands in which both gently and heavily used fluid increased in concentration in comparison to unused fluid. (**D**) Brands in which concentrations of both gently and heavily used fluid decreased in comparison to that of the unused fluid, and (**E**) Brands in which changes in element concentration relative to that of the unused were similar in the gently and heavily used products. Red squares = increase; blue squares = decrease; tan squares = no change.

The concentrations of elements in the unused and used fluids are compared for each brand of EC in Figure 2. Data are clustered in Figure 2A to show the products in which most elements increased after use. Four products (Greensmoke gently and heavily used, BluCig heavily used, and V2 Cig heavily used) had higher concentrations of most elements after use. In most other brands, element levels after use either increased or stayed the same, as in the unused fluid. The exceptions were gently used Safe Cig and both gently and heavily used NJOY NPRO, in which most elements decreased in concentration after use. The elements that frequently increased after use included some that are potentially harmful (zinc, nickel, copper, and lead). The elements that often did not change were in low concentrations (titanium, boron, cadmium, vanadium, and silver).

When comparing element concentrations in gently and heavily used fluids to the unused fluids within each brand, there were four patterns. (1) Concentrations were higher in heavily used than in gently used fluid, as seen with BluCig and BluCig Disposable (Figure 2B). (2) Concentrations in both the gently and heavily used fluids increased, as seen with Greensmoke (Figure 2C). (3) Both the gently and heavily used fluids decreased relative to the unused, as seen with NJOY NPRO and SafeCig (Figure 2D). (4) The individual element concentrations were similar between the gently and heavily used fluids, as seen with South Beach Smoke, V2 cigs, and Vype fluids (Figure 2E). There was only gently used fluid for SafeCig and Vype, the individual element concentrations in comparison to the unused decreased in SafeCig (Figure 2D), and the concentrations in the unused and gently used fluids were similar for Vype (Figure 2E).

The element concentrations in the fluid of the heavily used ECs were compared to fluid in the gently used products (Figure S23). In most comparisons (97), the element concentrations were higher in the heavily used group. In 41 comparisons, the concentrations were similar in the heavily and gently used groups. In 38 comparisons, the element concentrations were lower in the heavily used products.

### 3.5. Nicotine and Flavor Chemical Concentrations in Unused and Used E-Liquids

The concentrations of nicotine in aged unused and used e-liquids are summarized in Table 2. The concentrations of nicotine labeled on the packaging for all brands were either 16, 18, or 24 mg of nicotine. The concentration measured in the aged unused and used e-liquids varied among brands and were all lower than the labeled concentration. In most brands, the nicotine concentration measured after aging was 57–85% lower than the concentration on the label. However, in two brands (NJOY NPRO unused and SafeCig unused), the measured concentration was 98 to 100% lower than the labeled concentration.

**Figure 3:**
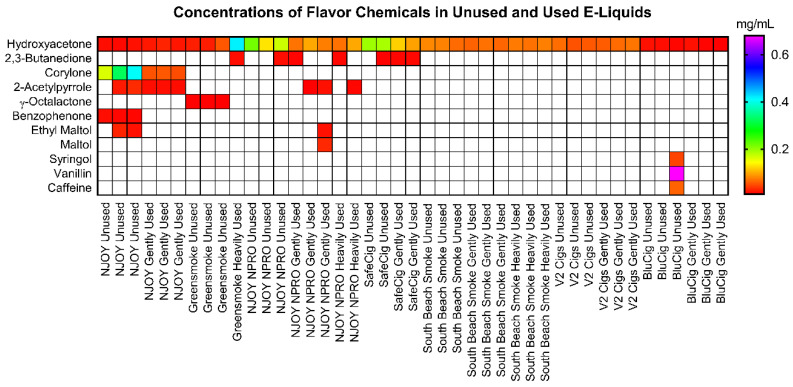
Heat map showing the concentration of flavor chemicals in aged unused and used e-liquids. Flavor chemicals are presented on the y axis, and EC products and sample types are on the *x* axis. All concentrations are in mg/mL White boxes indicate the flavor chemical was not detected.

A total of 33 flavor chemicals were identified in 10 brands of aged unused and used e-liquids. Twelve were above the limit of quantification (>0.01 mg/mL) and are shown in Figure 3. The 21 flavor chemicals that were below the limit of quantification are presented in Supplemental Table S9. On the *y* axis of the heatmap, the flavor chemicals are arranged by frequency in the unused and used e-liquids. The concentration of each flavor chemical ranged from 0.01 to 0.679 mg/mL (Figure 3). Hydroxyacetone was the only flavor chemical detected in all fluid samples (top of heat map). Hydroxyacetone (0.01 to 0.419 mg/mL), corylone (0.05 to 0.409 mg/mL), and vanillin (0.680 mg/mL) had the highest concentrations, but all were <1 mg/mL. 

### 3.6. pH and Acid Concentrations in Aged Unused E-Liquids

**Figure 4:**
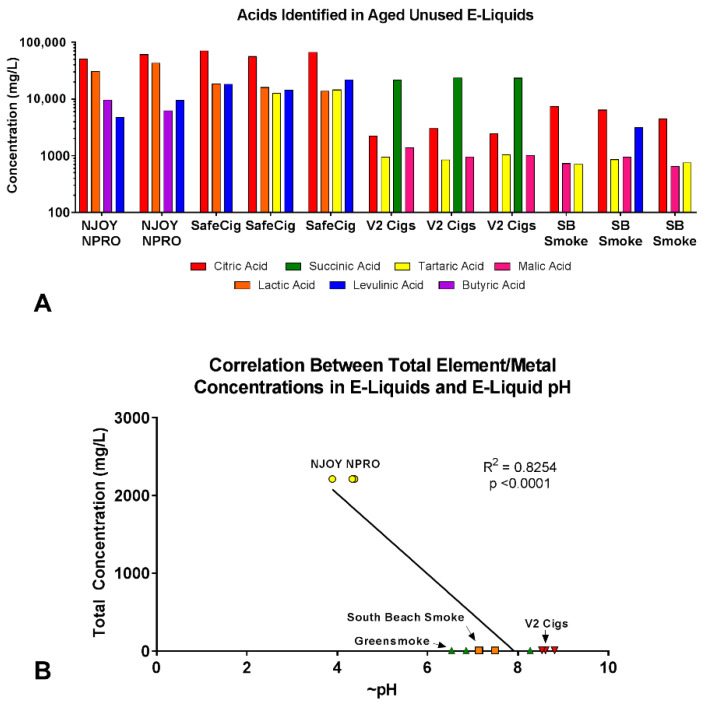
pH and acids in aged unused e-liquids. (**A**) Concentrations (mg/L) of acids measured in aged unused e-liquids. (**B**). Correlation graph showing relationship between total element/metal concentrations (mg/L) in the e-liquid and pH of the e-liquid.

To understand why NJOY NPRO and SafeCig had much higher total element/metal concentrations than other products, the acids in each product were identified and quantified (Figure 4A). Seven of eleven common organic acids examined (citric, lactic, succinic, levulinic, tartaric, butyric, malic) were present above the limit of quantification in at least one of the products (Figure 4A, Table S9). Citric acid was found in all e-liquid samples (concentrations ranged from 2205 to 70,317 mg/L) (Figure 4A). Citric, lactic, levulinic, tartaric, and butyric acids had the highest concentrations in NJOY NPRO and Safe Cig (range was from 4694 to 70,317 mg/L). In contrast, citric, succinic, levulinic, tartaric, and malic acid concentrations were relatively low in South Beach Smoke and V2 Cig (range was 647 to 23,295 mg/L) (Figure 4A).

To determine if pH affected total element/metal concentrations in aged unused e-liquids, linear regression was performed on the data (Figure 4B). The pH and total element/metal concentrations were highly correlated (R^2^ = 0.83, *p* < 0.0001) (Figure 4B). NJOY NPRO had the highest total concentration of elements/metals and also had the lowest pH (3.89–4.38) (Figure 4B). Greensmoke, South Beach Smoke, and V2 Cigs had overall lower total element/metal concentrations in their fluid and higher pHs (6.58–8.27, 7.13–7.49, and 8.53–8.81, respectively) (Figure 4B).

## 4. Discussion

This is the first study to evaluate the concentrations of elements/metals, nicotine, and flavor chemicals from first-generation ECs that were unused, gently used, or heavily used and stored for 5–10 years. The total concentration of elements/metals after 5–10 years of storage varied between brands and ranged from 5.65 mg/mL (Vuse) to 2214 mg/mL (NJOY NPRO). Copper, zinc, tin, nickel, and lead had the highest concentrations in e-liquids. In some brands, the concentrations of individual elements varied within the brand, e.g., nickel concentrations varied within heavily used South Beach Smoke, and copper varied with all samples of BluCig Disposables. Element concentrations generally, but not always, increased after use, and changes in concentrations after use were related to the brand and whether they were gently or heavily used. For example, Greensmoke, South Beach Smoke, and Vype often had higher elemental concentrations in gently/heavily used samples. The concentration of measured nicotine relative to the label concentration decreased in all brands, regardless of use, with some products having no quantifiable nicotine after 5–10 years of storage. Most products had few flavor chemicals that were low in concentration.

Two brands (NJOY NPRO, SafeCig) had the highest total element/metal concentrations in aged unused e-liquids. These brands also had the highest concentration of acids, causing their e-liquids to have low pHs. The e-liquid pH was highly correlated with total elements/metals in aged unused e-liquids. NJOY NPRO and Safe Cig (both purchased between 2012–2013) did not have benzoic acid in their fluid, but contained significant levels of other acids, showing that some manufacturers were using acids before JUUL introduced benzoic acid in their products [33]. Some acids (citric and lactic acid) are known to cause corrosion during storage [34] and are commercially used to etch metals [35,36], this could explain why NJOY NPRO and SafeCig had high total element/metal concentrations in the aged unused e-liquids than brands with lower levels of acid and higher pH levels. Inhalation of any of the acids present in this study can cause coughing, bronchoconstriction, and respiratory irritation [37,38,39]. These data are important for consumers as they will likely be exposed to higher concentrations of metals, when using products with low pHs.

*Metals increase*. In all brands, except NJOY NPRO, most elements/metals, including potassium, zinc, calcium, nickel, sodium, chromium, copper, magnesium, lead, and manganese, increased in the gently/heavily used e-liquid relative to the unused fluid. This is likely due to metals emitted from atomizer components during heating and being trapped in the e-liquid. Usually, concentrations were equivalent or higher in the heavily versus gently used samples, supporting the idea that increased use increases elemental concentrations in e-liquids. This increase with use was observed in disposable BluCig products, where concentrations of copper (not detected) and boron (0.041 µg/10 puffs), measured in the first 60 puffs, increased to 0.095 µg/10 puffs (copper) and 0.062 µg/10 puffs (boron) in puffs 120–180 [2]. The storage temperature could also affect the increase in metals in the fluid. In a recent study evaluating the metal concentrations of lead, nickel, and zinc in clearomizers, the concentrations of all three elements increased when the clearomizers were stored in a temperature range of 22–40° [40]. These increases in element/metal concentrations also varied with EC brand. These data indicate that exposure to aerosol metals increases with the use of an EC, a point that could be important in evaluating the health effects of metal exposure in EC users. Some of the elements that increased with use are potentially harmful (e.g., copper, zinc, nickel, chromium, manganese, and lead).

*Metals Decrease*. In two brands (NJOY NPRO, SafeCig), element concentrations were lower in the used fluids than the unused fluids. While this was unexpected, it clearly illustrated the complexities of working with ECs. It is possible that chelation or sorption of elements to the atomizer component(s) occurred during storage.

*Source of elements/metals in e-liquid.* The elements/metals in e-liquids come from two sources, namely the unused fluid and the atomizer components. These transfer into the fluid upon heating. Unused refill and e-liquid, which has not aged, contained selenium, tin, silicon, aluminum, calcium, sodium, and arsenic [11], while other labs have reported the presence of elements such as copper, chromium, manganese, nickel, lead, and zinc in unused commercial refill fluids [12,18]. These elements are likely introduced with the other fluid ingredients. Propylene glycol (PG) and glycerin (G) both contain most of these elements [11]. Our prior study showed that the concentration of selenium in PG and G is very similar across products [11]. In the current study, selenium was sometimes found at concentrations similar to those we reported previously (0.048–0.348 mg/L), and it did not change after use. This outcome would be expected, as selenium’s source is PG and G, not atomizer components. However, in some products, selenium decreased after use (e.g., BluCig) or was not detected in the unused fluid (e.g., Greensmoke). These data suggest that in some brands, selenium can be chelated after the EC has been used, that chelation may occur in some products before they are heated, or that some batches of PG and G have levels of selenium below the level of quantification. Selenium does transfer to the aerosol of V2 Cigs ECs and clearomizer/mod-style products [11] and is of concern as it is found on the FDA’s Harmful and Potentially Harmful Constituents in Tobacco Products and Tobacco Smoke list [7]. The other elements in unused e-liquids are generally present in lower concentrations than selenium or are not considered toxic (e.g., sodium). However, some, such as arsenic, could present a health concern.

Many of the elements/metals in e-liquids have been identified in the atomizing units of first-generation ECs (Table S8) [1,2,3] and increases in element concentrations after use are likely due to the release of atomizer elements during heating. As examples, the filaments and wires were alloys of chromium, nickel, copper, iron, aluminum (nichrome, kanthal, or elinvar); the wire joints were often tin or tin/lead solder, brass clamps (copper and zinc); the wicks were predominantly silicon, and contained calcium, magnesium, and aluminum; the air tubes were usually brass (copper, zinc) with nickel plating [1,2,3,6]. Some elements measured in the fluid (boron, cadmium, cobalt) have not been identified in the atomizer components. This could be because not all EC components have been analyzed (e.g., the mouthpiece shell, sealing caps, batteries, micro-processing chips, buttons, and adapters), or because electron microscopy and energy dispersive spectroscopy are a less sensitive detection methods than ICP-OES, or because these elements could be due to environmental contamination during storage.

*Nicotine and flavor chemicals*. The observed decrease in nicotine concentration in aged products versus label concentrations could be due to several factors. It is likely that some nicotine degraded during heating or evaporated during storage. Discrepancies have been reported between labeled and measured nicotine concentrations [21,41,42,43,44,45,46], and these are likely due to labeling errors, manufacturing errors or nicotine contamination. The discrepancies we observed were larger than would be expected for labeling errors [41,42,43,44] and were likely due to loss of nicotine from the ECs, degradation of nicotine over time, or poor packaging.

While many e-liquids have multiple flavor chemicals, often at high concentration [22,23,24,25,27], there were very low concentrations of flavor chemicals in the aged e-liquids. This could be because tobacco-flavored products produced 10 years ago generally had few flavor chemicals, and these were generally low in concentration [22,24,25,47]. The data in Figure 3 are consistent with low concentrations being used in early EC products, but could also indicate that there was degradation or evaporation during aging. Hydroxyacetone, which was present in all products, was likely a degradation product of the solvents [48,49,50]. Although flavor chemical concentrations are low, some of these chemicals may be harmful. For example, 2,3 butanedione can produce bronchiolitis obliterans or “popcorn-lung” [51], and γ-octalacetone is a respiratory irritant [52]. The concentrations of ethyl maltol, maltol, vanillin in the aged unused and used fluids were high enough to cause cytotoxicity to respiratory epithelium in the MTT assay [22,24,25].

*Human health concerns.* Some e-liquid elements/metals that we found are on the FDA and ATSDR’s harmful chemical lists (nickel, zinc, copper, selenium, lead, arsenic, cadmium, cobalt, chromium, manganese, aluminum, vanadium) [7,15], raising concern about their potential effects on health. Arsenic, cadmium, chromium, cobalt, lead, and nickel are carcinogens, while arsenic, cadmium, chromium, cobalt, lead, nickel, selenium affect the cardiovascular and respiratory systems. Most elements in e-liquids can cause skin irritation [9,10,53], and one case report found that high concentrations of nickel in e-liquid caused an EC consumer to develop contact dermatitis after spilling the fluid on her hand [54].

In most of our data, the concentration of toxic metals in e-liquids increased with use [11,12,18]. This raises the question: Should there be a limit on how many puffs are taken with an EC? Some early products were designed to deliver only 200 puffs (e.g., Vuse). However, there has been a trend toward larger tanks, more puffs, and repeated heating of atomizer components, all of which may contribute to raising element concentrations in fluid. The original fourth-generation products generally had small pods, which would deliver fewer puffs, although some of these are refillable and some newer models claim over 6000 puffs (e.g., Flum Pebble). Regulating the number of puffs/atomizer may help to reduce exposure to metals that originate from atomizer components.

The concentrations of eight elements (aluminum, arsenic, cadmium, cobalt, chromium, copper, iron, manganese, nickel, lead, zinc) were similar to the reported ranges measured in the fluids after use in cartridge and tank-style ECs [11,12,17]. However, six of the elements (aluminum, cobalt, copper, nickel, lead, zinc) were 3–5900 times higher in the aged fluids in the current study than in previous reports (Table S10), suggesting that these elements may have increased during aging. This raises the questions as to whether EC products should have limited shelf lives, whether these should be reported on packaging to reduce exposure to harmful metals, and as to what this shelf life should be?

*Environmental concerns*. First-generation ECs are discarded after use, which could contribute to environmental pollution, as has already been observed for conventional cigarette butt (CB) waste [55,56]. CB filters, which collect harmful chemicals in cigarette smoke, often end up in storm drains, wastewater treatment centers, rivers, streams, and on beaches, locations where chemicals can leach into the environment [57]. Extracts and leachates from CBs and first-generation ECs have toxic effects on microorganisms and wildlife [55,58,59,60,61,62], and produced teratogenic effects in Xenopus embryos, with ECs being less harmful than CBs in both studies [58]. The concentrations of metals (aluminum, cadmium, chromium, copper, iron, lead, manganese, nickel, titanium, zinc) in CB leachates increased over time. This suggested that the longer CBs and ECs (as seen in the current study) remain in the environment, the more metal contamination will occur [62]. The concentrations of copper, lead, nickel, and zinc were higher in the aged unused and used e-liquids than in unsmoked and smoked CB leachates (Table S11) [62], reflecting the difference in metal composition of these two types of tobacco products. These data are important, as the toxic metals and nicotine (a toxicant) from ECs and CBs could leach into the environment after disposal [61,63,64]. Our observed decrease in nicotine concentrations and low concentrations of flavor chemicals are consistent with losses from fluids during storage and suggest that discarded EC products could contribute to environmental pollution. More data are needed on the toxic effects of metals, nicotine, and organic chemicals in used ECs and their potential effects on the environment.

*EC waste regulation*. As EC use increases and conventional cigarette markets decline, there is a likelihood that EC pollution will become a major health and environmental problem that may surpass the current CB waste problem. However, under the National Environmental Policy Act by the Council on Environmental Quality Regulations, and Unfunded Mandates Reform Act of 1995, the US Food and Drug Administration (FDA) can issue an Environmental Impact Assessment to require that manufacturers take more responsibility for recycling or properly disposing of ECs [55,65,66]. This could be a positive step in preventing EC environmental contamination, protecting human and non-human health, as well as environmental resources.

## 5. Conclusions

In most ECs, elements/metals increased in e-liquids after heavy use, generally having higher concentrations of elements than those that were gently used. The high total element concentrations in some unused fluids suggest that elements leached into the fluid during storage, indicating a need for a better understanding of product shelf life. The lack of expiration dates on EC products, in conjunction with increases in toxic metals with use and storage, could cause adverse health effects in EC consumers. Nicotine was always found to be lower in concentration after storage than expected based on the label concentrations. Flavor chemical concentrations were always low after storage. This could be due to chemical loss during storage or the use of relatively low concentrations of flavor chemicals in first-generation tobacco-flavored products. These data indicate that future studies are needed to acquire a better understanding of chemical changes in fluids during storage and after the product has been discarded. This is needed to better evaluate the effects of EC products on both human and environmental health.

## Figures and Tables

**Table 1:** Brand, sample types, average individual element concentrations, and total concentrations (mg/L).

Brand (Sample Type)	Copper	Zinc	Tin	Nickel	Lead	Total ^a^
BluCig (Unused)	28.13 ± 9.00	2.23 ± 1.43	6.12 ± 6.43	1.60 ± 1.59	0.12 ± 0.12	44.86 ± 14.77
BluCig (Gently Used)	11.90 ± 3.37	4.94 ± 0.45	0.13 ± 0.08	0.25 ± 0.04	0.03 ± 0.01	22.89 ± 3.61
BluCig (Heavily Used)	118.49 ± 20.20	30.55 ± 15.19	5.92 ± 4.99	15.05 ± 7.62	2.53 ± 1.52	191.26 ± 33.92
BluCig Disposable (Unused)	44.92 ± 22.04	15.32 ± 5.85	0.05 ± 0.00	0.01 ± 0.00	0.03 ± 0.03	68.16 ± 24.97
BluCig Disposable (Gently Used)	44.94 ± 43.77	22.01 ± 24.39	0.03 ± 0.03	N/D	0.04 ± 0.04	73.28 ± 74.20
BluCig Disposable (Heavily Used)	98.67 ± 30.50	49.01 ± 15.14	0.05 ± 0.00	0.05 ± 0.07	0.09 ± 0.03	163.83 ± 49.83
Greensmoke (Unused)	4.44 ± 0.24	1.46 ± 0.22	0.05 ± 0.00	2.31 ± 2.40	N/D	12.31 ± 3.67
Greensmoke (Gently Used)	373.61 ± 49.19	295.79 ± 100.01	287.63 ± 129.41	41.99 ± 15.34	46.76 ± 22.18	1478.32 ± 468.04
Greensmoke (Heavily Used)	424.16 ± 174.56	271.66 ± 58.50	175.51 ± 70.59	71.09 ± 45.18	28.24 ± 38.85	1292.95 ± 130.38
NJOY NPRO (Unused)	1161.63 ± 509.25	275.19 ± 21.43	7.58 ± 5.14	63.87 ± 13.57	50.24 ± 37.35	2214.87 ± 852.53
NJOY NPRO (Gently Used)	255.55 ± 156.01	123.74 ± 58.61	1.52 ± 1.34	9.33 ± 4.21	16.91 ± 13.03	542.73 ± 315.95
NJOY NPRO (Heavily Used)	216.86 ± 187.43	93.35 ± 87.09	1.25 ± 1.36	13.40 ± 14.04	6.77 ± 7.78	441.06 ± 412.64
SafeCig (Unused)	909.35 ± 195.84	224.51 ± 28.93	108.20 ± 74.52	32.52 ± 21.93	32.79 ± 9.72	1661.18 ± 382.00
SafeCig (Gently Used)	333.10 ± 189.88	161.06 ± 69.44	74.52 ± 30.36	19.63 ± 7.87	6.31 ± 6.70	772.74 ± 390.48
MarkTen (Gently Used)	368.91 ± 181.67	285.82 ± 137.63	0.48 ± 0.05	0.07 ± 0.02	0.78 ± 0.44	916.17 ± 446.43
MarkTen (Heavily Used)	281.16 ± 115.73	210.87 ± 8.9	0.20 ± 0.08	0.08 ± 0.02	0.92 ± 0.35	671.35 ± 193.35
Vuse (Gently Used)	0.16 ± 0.16	0.51 ± 0.02	0.13 ± 0.07	0.24 ± 0.31	0.03 ± 0.02	5.65 ± 0.52
Vuse (Heavily Used)	0.59 ± 0.52	0.92 ± 0.30	0.10 ± 0.03	0.09 ± 0.01	0.02 ± 0.03	6.91 ± 0.46
South Beach Smoke (Unused)	2.39 ± 1.20	1.26 ± 0.28	0.10 ± 0.04	0.04 ± 0.00	0.01 ± 0.02	10.07 ± 2.44
South Beach Smoke (Gently Used)	3.24 ± 2.03	2.15 ± 1.23	0.10 ± 0.04	0.06 ± 0.02	0.05 ± 0.03	14.23 ± 4.43
South Beach Smoke (Heavily Used)	5.26 ± 5.29	4.74 ± 4.54	0.12 ± 0.08	0.53 ± 0.64	0.23 ± 0.31	35.80 ± 14.85
V2 Cig (Unused)	0.26 ± 0.08	0.32 ± 0.03	0.04 ± 0.00	0.03 ± 0.01	N/D	11.96 ± 4.77
V2 Cig (Gently Used)	1.90 ± 1.40	1.00 ± 0.79	0.05 ± 0.01	0.18 ± 0.05	0.01 ± 0.02	12.83 ± 2.84
V2 Cig (Heavily Used)	0.73 ± 0.38	0.78 ± 0.15	0.04 ± 0.00	0.25 ± 0.06	N/D	32.66 ± 5.81
Vype (Unused)	70.87 ± 1.76	33.30 ± 4.88	0.07 ± 0.02	0.01 ± 0.00	0.07 ± 0.01	117.53 ± 5.79
Vype (Gently Used)	88.55 ± 3.21	49.45 ± 0.41	0.18 ± 0.18	0.01 ± 0.00	0.10 ± 0.01	151.47 ± 0.13

^a^ Total concentration of all 22 elements measured in the fluids. Abbreviations: N/D; Not Detected, N/M; Not Measured.

**Table 2:** Nicotine concentrations in aged unused and used e-liquids.

Brand/Sample Type	EC Type	Nicotine Conc on Package (mg)	Nicotine Conc Measured (mg/mL)	% Difference ^a^
NJOY NPRO Unused	Cartomizer	18	0	−100
NJOY NPRO Unused	Cartomizer	18	0.1	−100
NJOY NPRO Unused	Cartomizer	18	0	−100
NJOY NPRO Gently Used	Cartomizer	18	2.6	−85
NJOY NPRO Gently Used	Cartomizer	18	4.5	−75
NJOY NPRO Gently Used	Cartomizer	18	4.4	−76
NJOY NPRO Heavily Used	Cartomizer	18	2.3	−87
NJOY NPRO Heavily Used	Cartomizer	18	7.8	−57
SafeCig Unused	Cartomizer	24	0.5	−98
SafeCig Unused	Cartomizer	24	0.3	−99
SafeCig Gently Used	Cartomizer	24	5.7	−76
SafeCig Gently Used	Cartomizer	24	5	−79
BluCig Unused	Disposable	24	8.2	−66
BluCig Unused	Disposable	24	7.4	−69
BluCig Unused	Disposable	24	11.6	−52
BluCig Gently Used	Disposable	24	6.4	−73
BluCig Gently Used	Disposable	24	4.7	−80
BluCig Gently Used	Disposable	24	8	−67
V2 Cigs Unused	Cartomizer	18	7.5	−58
V2 Cigs Unused	Cartomizer	18	9	−50
V2 Cigs Unused	Cartomizer	18	9.2	−49
V2 Cigs Gently Used	Cartomizer	18	8.3	−54
V2 Cigs Gently Used	Cartomizer	18	8.3	−54
V2 Cigs Gently Used	Cartomizer	18	8.2	−55
South Beach Smoke Unused	Cartomizer	16	7.1	−56
South Beach Smoke Unused	Cartomizer	16	6.6	−59
South Beach Smoke Unused	Cartomizer	16	6.5	−60
South Beach Smoke Gently Used	Cartomizer	16	7.1	−56
South Beach Smoke Gently Used	Cartomizer	16	7.1	−55
South Beach Smoke Gently Used	Cartomizer	16	6.8	−57
South Beach Smoke Heavily Used	Cartomizer	16	6.2	−61
South Beach Smoke Heavily Used	Cartomizer	16	5.3	−67
South Beach Smoke Heavily Used	Cartomizer	16	6.6	−59
Greensmoke Unused	Cartomizer	18	10.4	−42
Greensmoke Unused	Cartomizer	18	10.5	−42
Greensmoke Unused	Cartomizer	18	10.2	−43
Greensmoke Heavily Used	Cartomizer	18	0.1	−99
NJOY Unused	Cartridge	18	13	−28
NJOY Unused	Cartridge	18	11.4	−36
NJOY Unused	Cartridge	18	11.1	−38
NJOY Gently Used	Cartridge	18	8.8	−51
NJOY Gently Used	Cartridge	18	8.7	−51
NJOY Gently Used	Cartridge	18	8.8	−51

^a^ Color gradient: green indicates lowest percent difference between labeled and measured, while red indicates highest percent difference.

## Data Availability

All data are available in the manuscript.

## Supplementary Materials

The following supporting information can be downloaded at: https://www.mdpi.com/article/10.3390/ijerph192416931/s1. Table S1: Electronic Cigarette Selection and Materials list. Running conditions for inductively coupled plasma optical emission spectroscopy and gas chromatography mass spectroscopy. Tables S2–S4: Brand, sample types, average individual element concentrations and total concentrations (mg/L). Table S5. Individual element concentration ranges for aged unused and used electronic cigarette fluid. Table S6. Brands which had the elements with the highest concentrations. Table S7. Element, brand, sample type, highest concentration in alphabetical and decreasing order. Figures S1–S22: Twenty-two individual element concentrations in ten brands of unused, gently used, and heavily used first-generation e-liquid. Figure S23: Heat map showing element concentrations in heavily used EC fluids relative to the gently used ECs fluids. Table S8. Correlation between element detected in unused (U), gently Used (G), and heavily used (H) e-liquid and elements identified in EC atomizer components. Table S9. Flavor chemicals detected and acids in aged unused and used e-liquid that were below limit of quantification or not detected. Table S10. Concentrations of elements/metals reported in the fluid. Table S11. Comparison of element/metal concentrations (mg/L) in cigarette butts (CB) and EC cartomizers.
