# Supplementary material for: Chemical Elements, Flavor Chemicals, and Nicotine in Unused and Used Electronic Cigarettes Aged 5–10 Years and Effects of pH
Source: Int J Environ Res Public Health. 2022 Dec 16;19(24):16931. doi: 10.3390/ijerph192416931 (PMC9778798; doi:10.3390/ijerph192416931)
Supplement: Supplementary file 1 [file ijerph-19-16931-s001.zip › ijerph-2027574-supplementary.pdf]

Supplemental Material for

Chemical Elements, Flavor Chemicals, and Nicotine in Unused and Used Electronic Cigarettes Aged 5-10 Years and Effects of pH

Monique Williams[1], Wentai Luo[2], Kevin McWhirter[2], Omeka Ikegbu[1], and Prue Talbot[1,*]

[1] Department of Molecular, Cell, and Systems Biology, University of California, Riverside, CA 92521, USA

[2] Department of Chemistry and Department of Civil & Environmental Engineering, Portland State University, Portland, OR 97207, USA

* Correspondence: talbot@ucr.edu; Tel.: +1-951-850-7783

Table of Contents

Table S1: Electronic cigarette selection and materials list.

Running conditions for inductively coupled plasma optical emission spectroscopy and gas chromatography mass spectroscopy

Table S2-4. Brand, sample types, average individual element concentrations and total concentrations (mg/L)

Table S5. Individual element concentration ranges for aged unused and used electronic cigarette fluid

Table S6. Brands which had the elements with the highest concentrations.

Table S7. Element, brand, sample type, highest concentration in alphabetical and decreasing order.

Figures S1-22. Twenty-two individual element concentrations in ten brands of unused, gently used, and heavily used first generation EC fluid.

Figure S23. Heat map showing element concentrations in heavily used EC fluids relative to the gently used ECs fluids

Table S 8. Correlation between elements detected in unused (U), gently used (G), and heavily used (H) e-liquid and elements identified in EC atomizer components.

Table S9. Flavor chemicals detected and acids in aged unused and used e-liquid that were below limit of quantification or not detected

Table S10. Concentrations of elements/metals reported in the fluid (μg/mg)

Table S11. Comparison of element/metal concentrations (mg/L) in cigarette butts (CB) and EC cartomizers

Table S1: Electronic cigarette selection and materials list.

**EC Style**	**Brand**	**Manufacturer**
Cartomizer	BluCig (Tobacco and Menthol)	Lorillard Inc., Greensboro, NC
	Greensmoke	GreenSmoke LLC, Richmond, VA
	MarkTen	Altria Group, Inc., Richmond, VA
	NJOY NPRO	Sottera Inc., Scottsdale, AZ
	SafeCig	The Safe Cig LLC, Los Angeles, CA
	South Beach Smoke	South Beach Java LP, Wood Dale, IL
	V2 Cigs	VMR Products LLC., Miami, FL
	Vuse	Reynolds American, Inc., Winston-Salem, NC
Disposable	BluCig	Lorillard Inc., Greensboro, NC
	Vype	Reynolds American, Inc., Winston-Salem, NC
Nitric Acid	Veritas Nitric Acid Redistilled	GFS Chemical Inc, Columbus, OH
Deionized water	Milli-Q	EMD Millipore Sigma, Burlington, MA
Element Standards	Aluminum	All elemental standards are from
	Arsenic	Inorganic Ventures, Christiansburg, VA
	Boron	
	Cadmium	
	Calcium	
	Chromium	
	Cobalt	
	Copper	
	Iron	
	Lead	
	Magnesium	
	Manganese	
	Nickel	
	Potassium	
	Selenium	
	Silicon	
	Silver	
	Sodium	
	Tin	
	Titanium	
	Vanadium	
	Zinc	
Flavor chemical analysis	Isopropyl alcohol	Fisher Scientific, Fair Lawn, NJ

Running conditions for inductively coupled plasma optical emission spectroscopy and gas chromatography/ mass spectroscopy

Inductively Coupled Plasma Optical Emission Spectroscopy (ICP-OES)

All metal analysis was performed using a Perkin–Elmer Optima 7300 DV (Perkin Elmer, USA) ICP-OES equipped with an autosampler, a nebulizer (Perkin-Elmer, N0777036 REV A, Cyclonic spray chamber Optima 5300DV, Quartz 7mm baffle drain line) and a segmented array detector charge coupled device detector (SCD) were used for all analyses. The ICP-OES was calibrated daily using four Perkin-Elmer multi-element calibration standards (Plus #2, #3, #4, and #5). NIST standard reference materials (Ultra Scientific, trace metal sample, catalogue number QCI-700A North Kingstown, RI) were used for quality control checks on the ICP calibrations. Running conditions for the ICP OES are as follows to increase stability: plasma flow = 15 L/min, auxiliary flow 0.2 L/min, nebulizer flow of 0.5 L/min, radio frequency power 1350 W, sample flow rate = 0.80 mL/min, and a read delay time of 12 sec. Yttrium at 2.5 ppm was used as an internal standard and was run in line with sample introduction into the nebulizer. 1% nitric acid and distilled deionized water were ran as a blank. When interference was observed for any element, additional peaks were monitored to identify the best wavelength for quantification.

Gas Chromatography Mass Spectrometry (GC-MS)

All flavor chemicals and nicotine analysis were performed using an Agilent 5975C GCMS system (Santa Clara, CA). The GC column was a Restek Rxi-624Sil MS (Bellefonte, PA, 30 m long, 0.25 mm id, and 1.4 µm film thickness). Twenty µL of 2000 ng/µL 1,2,3-trichlorobenzene as internal standard was added to each sample before GC/MS analysis. One µL of each sample was injected into the GC injector with a 10:1 split. The injector temperature was 235 °C. The GC temperature program for all analyses was: 40 °C hold for 2 min; 10 °C/min to 100 °C; then 12 °C/min to 280 °C; hold for 8 min at 280 °C; then 10 °C/min to 230 °C. The MS was operated in electron impact ionization mode at 70 eV in positive ion mode. The ion source temperature was 220 °C. The scan range was 34 to 400 amu. Each target analyte was quantitated using authentic standards and an internal standard of 1,2,3-trichlorobenzene normalized multipoint calibration. All reported concentration values were based on a 1:20 dilution, except for overloaded peaks at 1:20 dilution, in which case quantitation was based on a 1:200 dilution sample.

Table S2. Brand, sample types, average individual element concentrations and total concentrations (mg/L)

Brand (Sample Type)	Sodium	Calcium	Silicon	Iron	Aluminum	Potassium	Total[a]
BluCig (Unused)	1.99 ± 0.74	1.64 ± 0.25	1.44 ± 0.25	0.45 ± 0.15	0.04 ± 0.00	N/M	44.86 ± 14.77
BluCig (Gently Used)	2.47 ± 0.09	0.53 ± 0.41	0.81 ± 0.03	0.80 ± 0.08	0.03 ± 0.01	0.08 ± 0.18	22.89 ± 3.61
BluCig (Heavily Used)	5.31 ± 0.33	8.08 ± 1.48	1.75 ± 0.27	1.43 ± 0.45	0.13 ± 0.08	0.54 ± 0.27	191.26 ± 33.92
BluCig Disposable (Unused)	2.12 ± 0.75	1.02 ± 0.17	1.83 ± 0.27	0.10 ± 0.05	0.11 ± 0.14	1.89 ± 2.90	68.16 ± 24.97
BluCig Disposable (Gently Used)	1.49 ± 1.29	2.61 ± 3.65	1.56 ± 1.38	0.03 ± 0.03	0.01 ± 0.01	0.12 ± 0.10	73.28 ± 74.20
BluCig Disposable (Heavily Used)	3.35 ± 1.27	9.01 ± 2.45	2.29 ± 0.37	0.10 ± 0.01	0.04 ± 0.01	0.24 ± 0.01	163.83 ± 49.83
Greensmoke (Unused)	0.74 ± 0.38	0.16 ± 0.04	2.65 ± 0.26	0.25 ± 0.17	0.02 ± 0.01	0.03 ± 0.03	12.31 ± 3.67
Greensmoke (Gently Used)	421.52 ± 244.40	0.48 ± 0.07	2.85 ± 0.38	3.36 ± 1.54	2.43 ± 2.74	0.59 ± 0.22	1478.32 ± 468.04
Greensmoke (Heavily Used)	310.59 ± 121.12	1.98 ± 1.28	2.68 ± 0.74	3.03 ± 3.29	1.09 ± 1.59	1.05 ± 0.66	1292.95 ± 130.38
NJOY NPRO (Unused)	644.05 ± 292.65	2.52 ± 0.28	3.28 ± 0.43	2.26 ± 0.64	2.92 ± 0.57	0.29 ± 0.10	2214.87 ± 852.53
NJOY NPRO (Gently Used)	128.55 ± 87.68	1.00 ± 0.31	4.21 ± 0.38	0.12 ± 0.08	0.39 ± 0.33	0.91 ± 0.08	542.73 ± 315.95
NJOY NPRO (Heavily Used)	102.31 ± 115.47	1.10 ± 0.49	4.08 ± 0.21	0.17 ± 0.20	0.25 ± 0.26	0.91 ± 0.43	441.06 ± 412.64
SafeCig (Unused)	344.65 ± 130.93	2.16 ± 0.25	0.63 ± 0.06	2.29 ± 0.99	1.98 ± 0.48	0.95 ± 0.14	1661.18 ± 382.00
SafeCig (Gently Used)	169.14 ± 86.67	2.52 ± 0.73	4.06 ± 0.07	0.35 ± 0.37	0.48 ± 0.32	0.51 ± 0.14	772.74 ± 390.48
MarkTen (Gently Used)	252.05 ± 123.29	5.59 ± 3.43	1.45 ± 0.47	0.05 ± 0.05	N/D	N/D	916.17 ± 446.43
MarkTen (Heavily Used)	171.04 ± 36.72	4.71 ± 2.15	1.29 ± 0.35	0.12 ± 0.02	N/D	N/D	671.35 ± 193.35
Vuse (Gently Used)	N/D	1.42 ± 0.14	1.53 ± 0.13	1.01 ± 0.26	N/D	N/D	5.65 ± 0.52
Vuse (Heavily Used)	0.03 ± 0.05	2.00 ± 0.45	1.34 ± 0.19	0.94 ± 0.44	0.05 ± 0.06	N/D	6.91 ± 0.46
South Beach Smoke (Unused)	0.99 ± 0.15	0.81 ± 0.86	4.00 ± 0.31	0.09 ± 0.03	0.01 ± 0.01	0.02 ± 0.01	10.07 ± 2.44
South Beach Smoke (Gently Used)	1.44 ± 0.21	2.54 ± 0.87	4.12 ± 0.38	0.07 ± 0.01	0.02 ± 0.01	0.16 ± 0.06	14.23 ± 4.43
South Beach Smoke (Heavily Used)	1.67 ± 0.16	18.42 ± 4.32	4.23 ± 0.31	0.08 ± 0.01	0.02 ± 0.01	0.10 ± 0.08	35.80 ± 14.85
V2 Cig (Unused)	1.37 ± 0.08	0.22 ± 0.06	9.30 ± 4.68	0.03 ± 0.00	0.01 ± 0.01	0.09 ± 0.02	11.96 ± 4.77
V2 Cig (Gently Used)	1.78 ± 0.27	0.32 ± 0.09	6.98 ± 0.24	0.03 ± 0.01	0.01 ± 0.01	0.24 ± 0.05	12.83 ± 2.84
V2 Cig (Heavily Used)	2.40 ± 0.67	18.45 ± 4.81	8.73 ± 0.71	0.08 ± 0.03	0.01 ± 0.00	0.22 ± 0.15	32.66 ± 5.81
Vype (Unused)	4.51 ± 0.29	5.11 ± 0.09	2.56 ± 0.16	0.02 ± 0.00	0.03 ± 0.00	0.19 ± 0.01	117.53 ± 5.79
Vype (Gently Used)	5.17 ± 0.13	4.94 ± 3.29	1.65 ± 0.65	0.03 ± 0.00	0.05 ± 0.01	0.24 ± 0.02	151.47 ± 0.13

[a]Total concentration of all 22 elements measured in the fluid. Abbreviations: N/D; Not Detected, N/M; Not Measured

Table S3. Brand, sample types, average individual element concentrations and total concentrations (mg/L)

Brand (Sample Type)	Boron	Magnesium	Manganese	Selenium	Chromium	Silver
BluCig (Unused)	0.25 ± 0.08	0.77 ± 0.02	0.03 ± 0.01	N/D	0.02 ± 0.02	0.01 ± 0.00
BluCig (Gently Used)	0.11 ± 0.02	0.72 ± 0.19	0.07 ± 0.02	N/D	0.01 ± 0.01	0.01 ± 0.00
BluCig (Heavily Used)	0.25 ± 0.06	0.87 ± 0.16	0.10 ± 0.01	N/D	0.19 ± 0.09	0.02 ± 0.01
BluCig Disposable (Unused)	0.13 ± 0.02	0.43 ± 0.38	0.011 ± 0.01	0.18 ± 0.02	N/D	0.01 ± 0.00
BluCig Disposable (Gently Used)	0.08 ± 0.07	0.20 ± 0.18	0.01 ± 0.02	0.12 ± 0.12	N/D	0.01 ± 0.00
BluCig Disposable (Heavily Used)	0.22 ± 0.09	0.48 ± 0.14	0.05 ± 0.01	0.15 ± 0.05	0.01 ± 0.01	0.01 ± 0.00
Greensmoke (Unused)	0.12 ± 0.02	0.08 ± 0.01	0.02 ± 0.02	N/D	0.01 ± 0.01	N/D
Greensmoke (Gently Used)	0.57 ± 0.04	0.11 ± 0.01	0.48 ± 0.36	N/D	0.03 ± 0.01	0.05 ± 0.01
Greensmoke (Heavily Used)	0.89 ± 0.43	0.34 ± 0.20	0.36 ± 0.37	N/D	0.16 ± 0.15	0.06 ± 0.03
NJOY NPRO (Unused)	0.20 ± 0.00	0.40 ± 0.06	0.10 ± 0.03	0.01 ± 0.01	0.04 ± 0.03	0.12 ± 0.03
NJOY NPRO (Gently Used)	0.10 ± 0.01	0.11 ± 0.04	0.14 ± 0.11	0.04 ± 0.01	0.05 ± 0.05	0.03 ± 0.02
NJOY NPRO (Heavily Used)	0.12 ± 0.01	0.13 ± 0.04	0.16 ± 0.17	0.05 ± 0.01	0.11 ± 0.11	0.03 ± 0.02
SafeCig (Unused)	0.18 ± 0.03	0.22 ± 0.01	0.47 ± 0.20	0.06 ± 0.01	0.04 ± 0.01	0.10 ± 0.01
SafeCig (Gently Used)	0.17 ± 0.06	0.33 ± 0.11	0.39 ± 0.23	0.07 ± 0.01	0.02 ± 0.01	0.04 ± 0.02
MarkTen (Gently Used)	0.40 ± 0.14	0.57 ± 0.21	0.02 ± 0.01	N/D	N/D	N/D
MarkTen (Heavily Used)	0.41 ± 0.00	0.52 ± 0.04	0.03 ± 0.00	N/D	N/D	N/D
Vuse (Gently Used)	0.13 ± 0.03	0.41 ± 0.03	0.02 ± 0.00	N/D	0.06 ± 0.05	N/D
Vuse (Heavily Used)	0.22 ± 0.10	0.55 ± 0.10	0.02 ± 0.01	N/D	0.04 ± 0.01	N/D
South Beach Smoke (Unused)	0.09 ± 0.02	0.09 ± 0.00	0.08 ± 0.08	0.08 ± 0.01	0.01 ± 0.00	N/M
South Beach Smoke (Gently Used)	0.07 ± 0.02	0.12 ± 0.02	0.02 ± 0.00	0.08 ± 0.00	N/D	N/M
South Beach Smoke (Heavily Used)	0.08 ± 0.01	0.18 ± 0.03	0.02 ± 0.01	0.08 ± 0.00	0.01 ± 0.00	N/M
V2 Cig (Unused)	0.10 ± 0.03	0.10 ± 0.00	0.02 ± 0.01	0.08 ± 0.01	N/D	N/M
V2 Cig (Gently Used)	0.10 ± 0.01	0.13 ± 0.02	0.01 ± 0.01	0.08 ± 0.00	0.01 ± 0.00	N/M
V2 Cig (Heavily Used)	0.16 ± 0.03	0.57 ± 0.15	0.14 ± 0.21	0.09 ± 0.02	0.01 ± 0.01	N/M
Vype (Unused)	0.30 ± 0.03	0.19 ± 0.01	N/D	0.30 ± 0.03	N/D	0.01 ± 0.00
Vype (Gently Used)	0.54 ± 0.22	0.18 ± 0.10	N/D	0.37 ± 0.01	N/D	0.01 ± 0.00

Abbreviations: N/D; Not Detected, N/M; Not Measured

Table S4. Brand, sample types, average individual element concentrations and total concentrations (mg/L)

Brand (Sample Type)	Cobalt	Titanium	Cadmium	Arsenic	Vanadium
BluCig (Unused)	N/D	0.03 ± 0.04	N/D	N/D	N/D
BluCig (Gently Used)	N/D	N/D	N/D	N/D	N/D
BluCig (Heavily Used)	N/D	0.03 ± 0.03	N/D	N/D	N/D
BluCig Disposable (Unused)	N/D	0.00 ± 0.01	N/D	N/M	N/D
BluCig Disposable (Gently Used)	N/D	0.01 ± 0.01	N/D	N/M	N/D
BluCig Disposable (Heavily Used)	N/D	0.02 ± 0.01	N/D	N/M	N/D
Greensmoke (Unused)	N/D	N/D	N/D	N/D	N/D
Greensmoke (Gently Used)	0.02 ± 0.02	N/D	0.06 ± 0.01	N/D	N/D
Greensmoke (Heavily Used)	0.04 ± 0.04	0.01 ± 0.00	0.03 ± 0.03	N/D	N/D
NJOY NPRO (Unused)	0.02 ± 0.01	0.05 ± 0.01	0.03 ± 0.02	N/D	N/D
NJOY NPRO (Gently Used)	0.01 ± 0.01	N/D	0.02 ± 0.01	N/D	N/D
NJOY NPRO (Heavily Used)	0.01 ± 0.01	N/D	0.01 ± 0.01	N/D	N/D
SafeCig (Unused)	0.04 ± 0.02	N/D	0.06 ± 0.01	N/D	N/D
SafeCig (Gently Used)	0.06 ± 0.04	N/D	N/D	N/D	N/D
MarkTen (Gently Used)	N/D	N/D	N/D	N/D	N/D
MarkTen (Heavily Used)	N/D	N/D	N/D	N/D	N/D
Vuse (Gently Used)	N/D	N/D	N/D	N/D	N/D
Vuse (Heavily Used)	N/D	N/D	N/D	N/D	N/D
South Beach Smoke (Unused)	N/D	N/D	N/D	N/D	N/D
South Beach Smoke (Gently Used)	N/D	N/D	N/D	N/D	N/D
South Beach Smoke (Heavily Used)	N/D	N/D	N/D	N/D	N/D
V2 Cig (Unused)	N/D	N/D	N/D	N/D	N/D
V2 Cig (Gently Used)	N/D	N/D	N/D	N/D	N/D
V2 Cig (Heavily Used)	N/D	N/D	N/D	N/D	N/D
Vype (Unused)	N/D	0.01 ± 0.00	N/D	N/M	N/D
Vype (Gently Used)	N/D	0.01 ± 0.01	N/D	N/M	N/D

Abbreviations: N/D; Not Detected, N/M; Not Measured

Table S5. Individual element concentration ranges for aged unused and used electronic cigarette fluid

Element	Overall Range (mg/L)	Unused Range (mg/L)	Gently Used Range (mg/L	Heavily Used Range (mg/L
Aluminum	0.008-5.466	0.013-4.831	0.011-5.466	0.008-2.924
Arsenic	0.009	0.009	ND	ND
Boron	0.059-1.266	0.071-0.338	0.059-0.694	0.068-1.266
Cadmium	0.001-0.078	0.008-0.078	0.001-0.073	0.004-0.063
Calcium	0.112-23.529	0.112-5.199	0.215-9.512	0.632-23.529
Chromium	0.003-0.323	0.003-0.075	0.003-0.117	0.004-0.323
Cobalt	0.003-0.106	0.014-0.065	0.003-0.106	0.005-0.070
Copper	0.050-1749.64	0.172-1749.64	0.050-572.35	0.077-608.20
Iron	0.022-6.82	0.022-5.728	0.024-4.405	0.031-6.821
Lead	0.011-93.38	0.011-93.38	0.012-72.33	0.036-72.90
Magnesium	0.067-1.051	0.067-0.874	0.085-0.934	0.097-1.05
Manganese	0.002-0.790	0.002-0.701	0.003-0.779	0.010-0.790
Nickel	0.001-102.49	0.001-78.289	0.002-54.307	0.002-102.49
Potassium	0.007-5.229	0.007-5.229	0.115-0.981	0.053-1.467
Selenium	0.015-0.372	0.015-0.328	0.039-0.372	0.048-0.199
Silicon	0.450-12.64	0.450-12.647	0.783-7.22	1.058-9.478
Silver	0.003-0.154	0.003-0.154	0.004-0.059	0.005-0.082
Sodium	0.091-979.46	0.466-979.46	1.214-692.17	0.091-412.44
Tin	0.033-420.41	0.036-200.92	0.036-420.41	0.033-248.38
Titanium	0.001-0.090	0.001-0.090	0.001-0.021	0.002-0.060
Vanadium	0.003-0.005	0.005	ND	0.003
Zinc	0.282-528.67	0.282-528.67	0.317-435.48	0.612-334.79

Table S6. Brands which had the elements with the highest concentrations

Brand	Elements with the Highest Concentrations
Greensmoke	Al, B, Cr, Fe, Mn, Ni, Sn
NJOY NPRO	Cu, Pb, Ag, Na, Zn
BluCig	Mg, Ti
BluCig D	K, V
SafeCig	Cd, Co
V2 Cigs	Ca, Si
Vype	As, Se

Table S7. Element, brand, sample type, highest concentration in decreasing order.

Element	Brand	Sample Type	Highest Conc (mg/L)
Copper	NJOY NPRO	Unused - 3	1749.64
Sodium	NJOY NPRO	Unused - 3	979.46
Zinc	NJOY NPRO	Unused - 2	528.67
Tin	Greensmoke	Gently Used - 1	420.41
Nickel	Greensmoke	Heavily Used - 1	102.49
Lead	NJOY NPRO	Unused - 3	93.38
Calcium	V2 Cigs	Heavily Used - 1	23.529
Silicon	V2 Cigs	Unused - 3	12.64
Iron	Greensmoke	Heavily Used - 1	6.82
Aluminum	Greensmoke	Gently Used - 3	5.466
Potassium	BluCig D	Unused - 3	5.229
Boron	Greensmoke	Heavily Used - 2	1.266
Magnesium	BluCig	Overtime - 1	1.051
Manganese	Greensmoke	Heavily Used - 1	0.79
Selenium	Vype	Gently Used - 2	0.372
Chromium	Greensmoke	Heavily Used - 1	0.323
Silver	NJOY NPRO	Unused - 3	0.154
Cobalt	SafeCig	Gently Used - 2	0.106
Titanium	BluCig	Unused - 2	0.09
Cadmium	SafeCig	Unused - 2	0.078
Arsenic	Vype	Unused - 1	0.009
Vanadium	BluCig D	Unused - 2	0.005

red = elements with highest concentrations, orange = divalent elements, yellow = most toxic, blue = similar in concentration, green = elements with lowest concentrations, grey = infrequently found

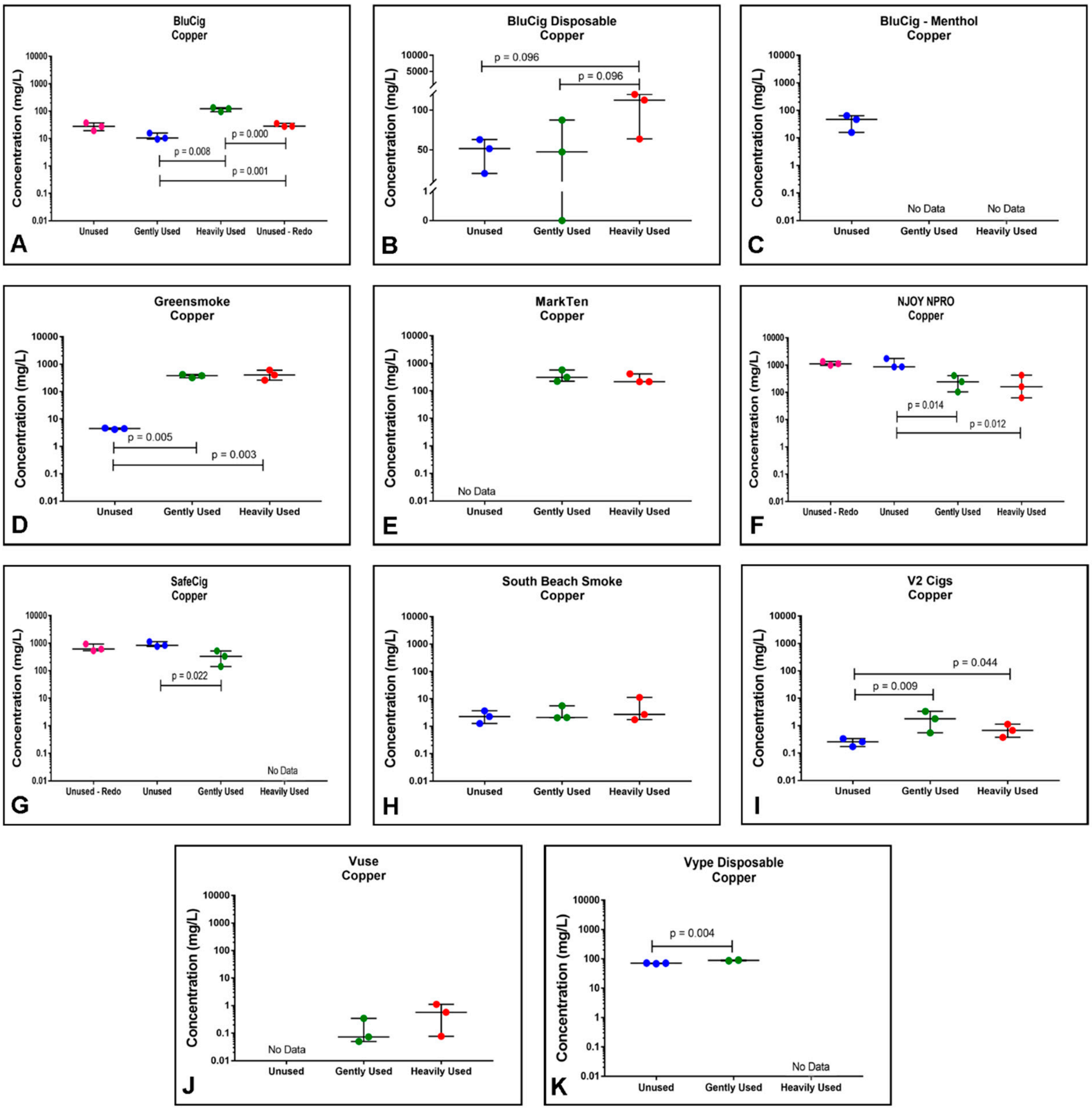

Figure S1. Copper concentrations in ten brands of unused, gently used, and heavily used first generation EC fluid. (A) BluCig, (B) BluCig Disposable, (C) BluCig – Menthol, (D) Greensmoke, (E) MarkTen, (F) NJOY NPRO, (G) SafeCig, (H) South Beach Smoke, (I) V2 Cigs, (J) Vuse, and (K) Vype Disposable. All fluids were analyzed in triplicates. Blue = unused, green = gently used, red = heavily used, pink = retesting of unused fluid.

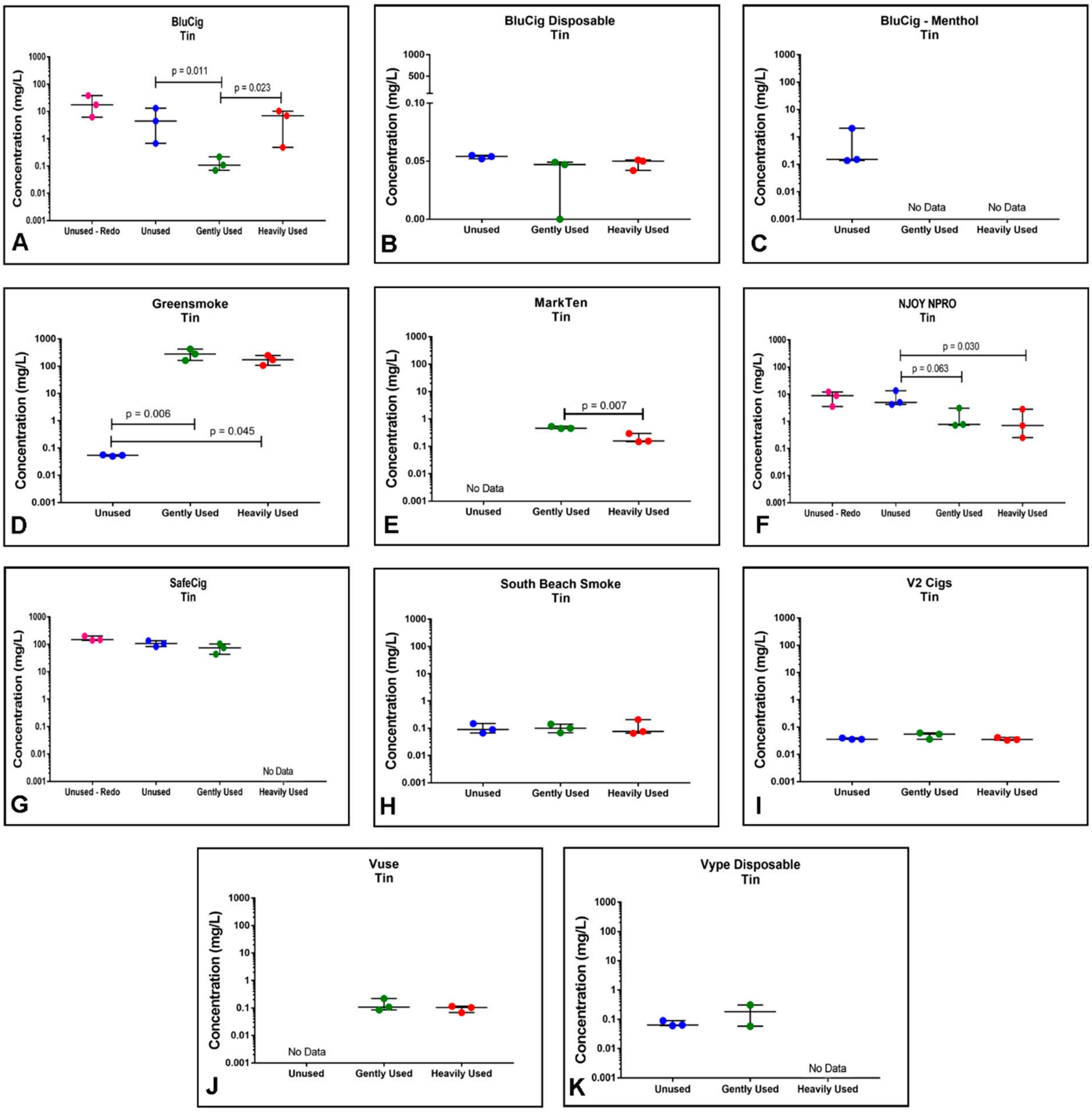

Figure S2. Tin concentrations in ten brands of unused, gently used, and heavily used first generation EC fluid. (A) BluCig, (B) BluCig Disposable, (C) BluCig – Menthol, (D) Greensmoke, (E) MarkTen, (F) NJOY NPRO, (G) SafeCig, (H) South Beach Smoke, (I) V2 Cigs, (J) Vuse, and (K) Vype Disposable. All fluids were analyzed in triplicates. Blue = unused, green = gently used, red = heavily used, pink = retesting of unused fluid.

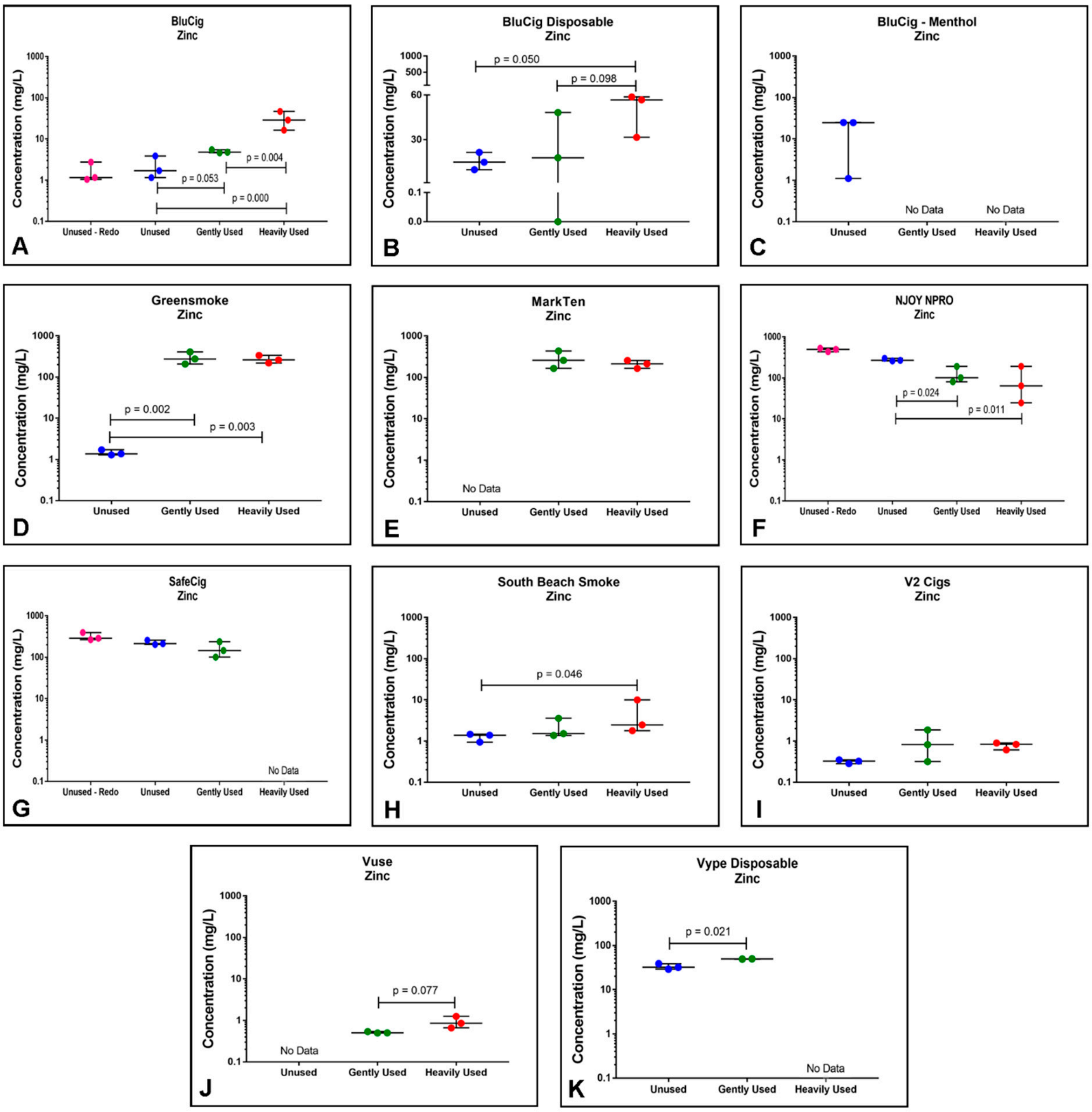

Figure S3. Zinc concentrations in ten brands of unused, gently used, and heavily used first generation EC fluid. (A) BluCig, (B) BluCig Disposable, (C) BluCig – Menthol, (D) Greensmoke, (E) MarkTen, (F) NJOY NPRO, (G) SafeCig, (H) South Beach Smoke, (I) V2 Cigs, (J) Vuse, and (K) Vype Disposable. All fluids were analyzed in triplicates. Blue = unused, green = gently used, red = heavily used, pink = retesting of unused fluid.

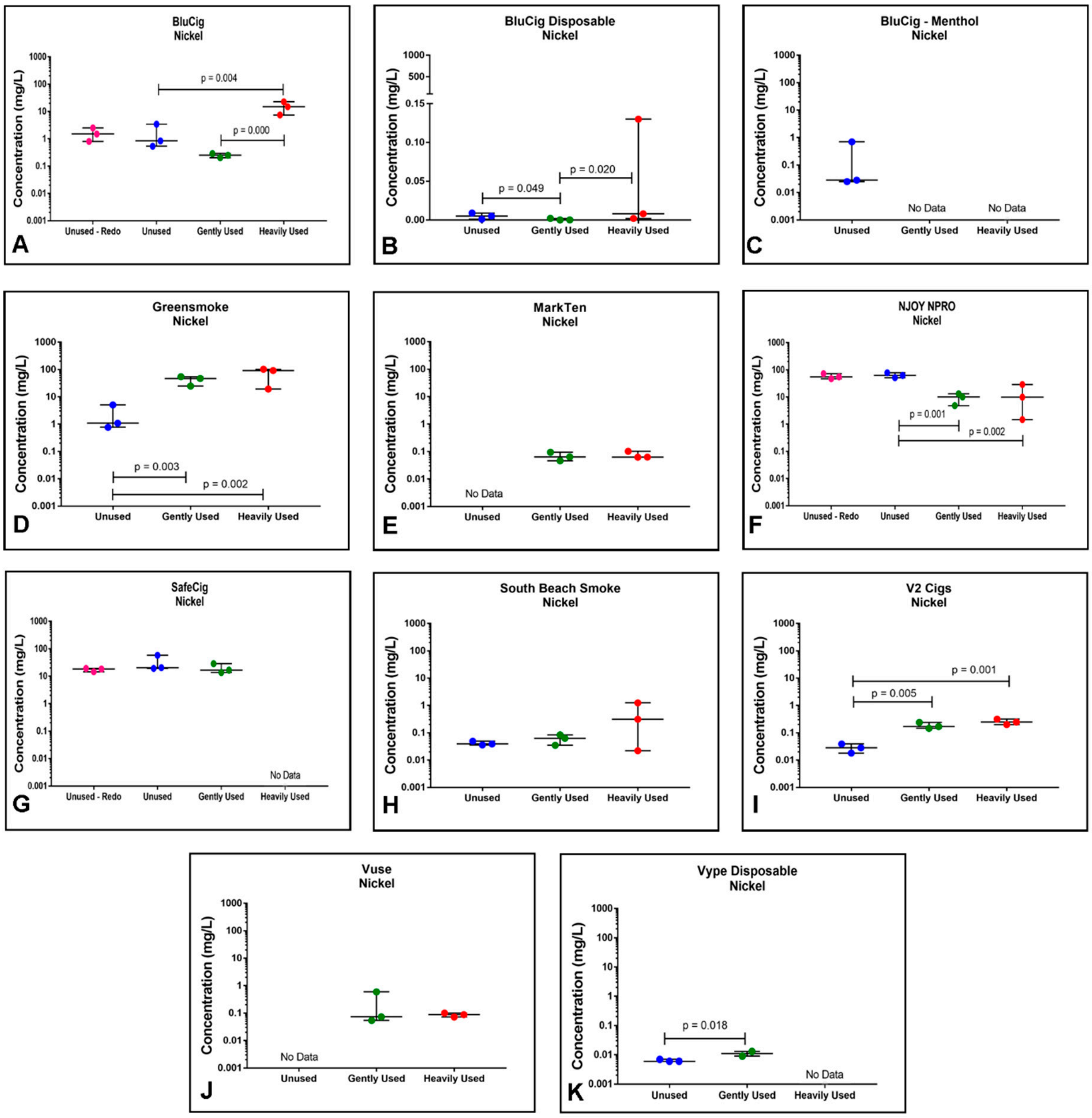

Figure S4. Nickel concentrations in ten brands of unused, gently used, and heavily used first generation EC fluid. (A) BluCig, (B) BluCig Disposable, (C) BluCig – Menthol, (D) Greensmoke, (E) MarkTen, (F) NJOY NPRO, (G) SafeCig, (H) South Beach Smoke, (I) V2 Cigs, (J) Vuse, and (K) Vype Disposable. All fluids were analyzed in triplicates. Blue = unused, green = gently used, red = heavily used, pink = retesting of unused fluid.

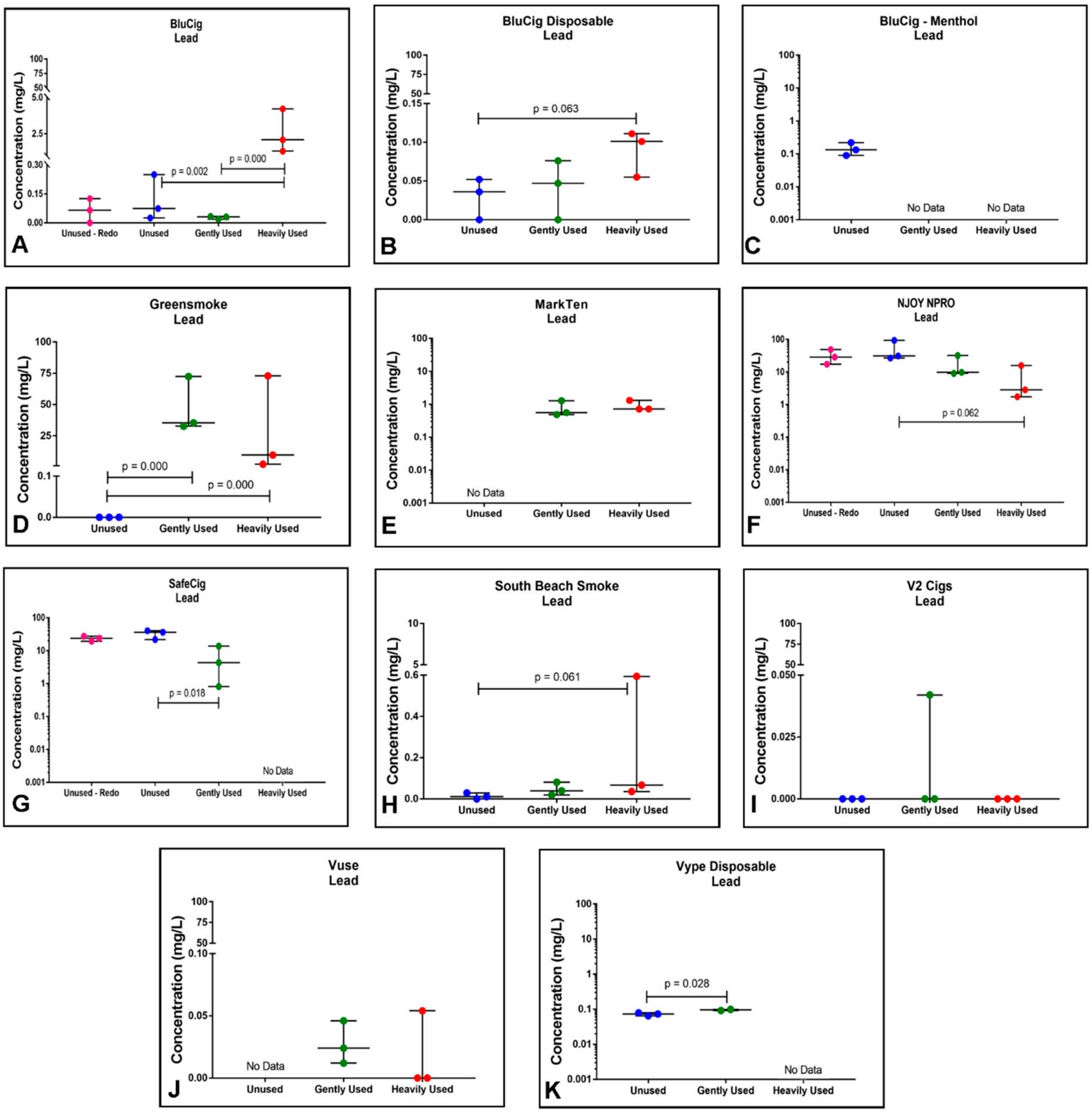

Figure S5. Lead concentrations in ten brands of unused, gently used, and heavily used first generation EC fluid. (A) BluCig, (B) BluCig Disposable, (C) BluCig – Menthol, (D) Greensmoke, (E) MarkTen, (F) NJOY NPRO, (G) SafeCig, (H) South Beach Smoke, (I) V2 Cigs, (J) Vuse, and (K) Vype Disposable. All fluids were analyzed in triplicates. Blue = unused, green = gently used, red = heavily used, pink = retesting of unused fluid.

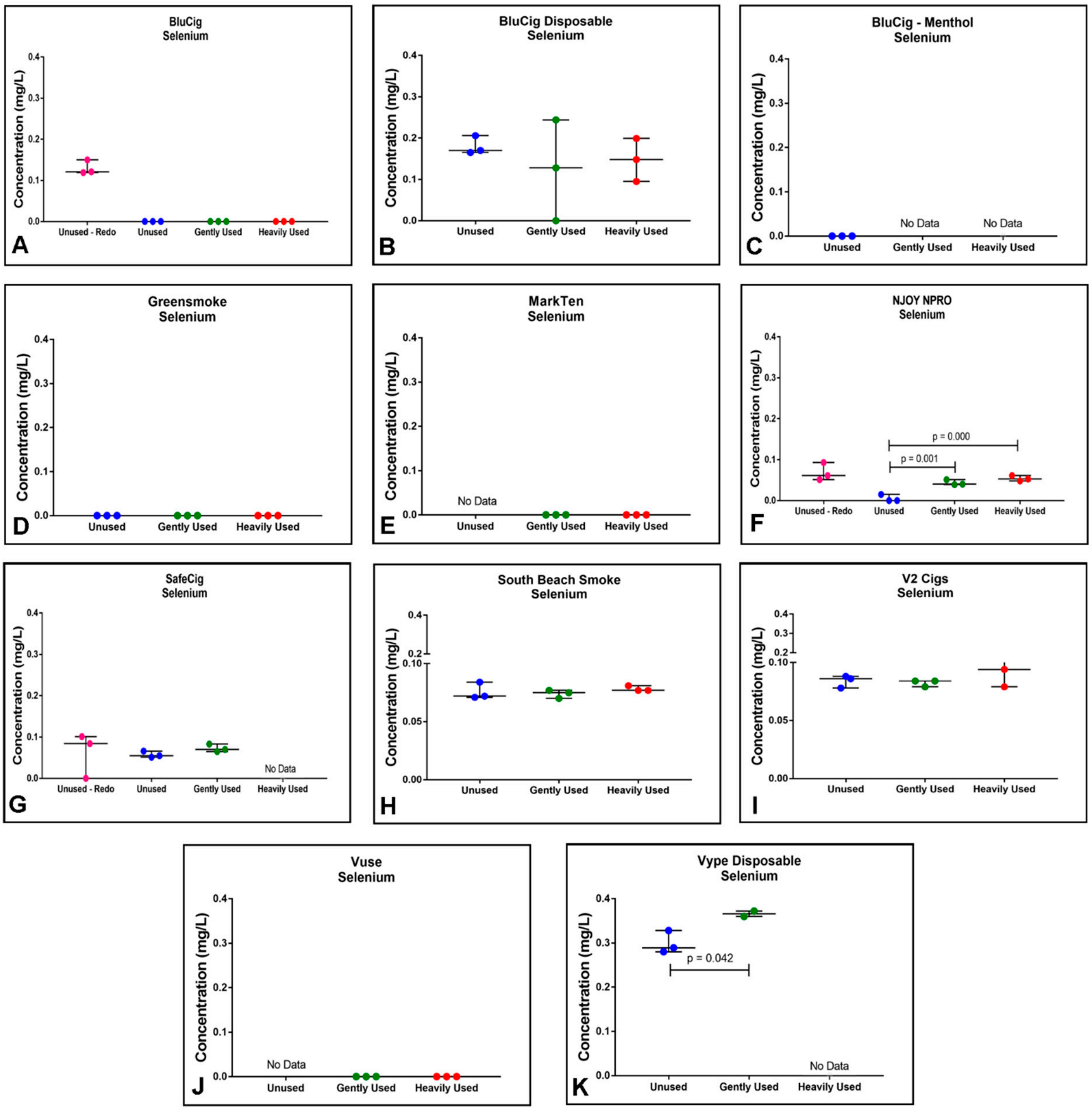

Figure S6. Selenium concentrations in ten brands of unused, gently used, and heavily used first generation EC fluid. (A) BluCig, (B) BluCig Disposable, (C) BluCig – Menthol, (D) Greensmoke, (E) MarkTen, (F) NJOY NPRO, (G) SafeCig, (H) South Beach Smoke, (I) V2 Cigs, (J) Vuse, and (K) Vype Disposable. All fluids were analyzed in triplicates. Blue = unused, green = gently used, red = heavily used, pink = retesting of unused fluid.

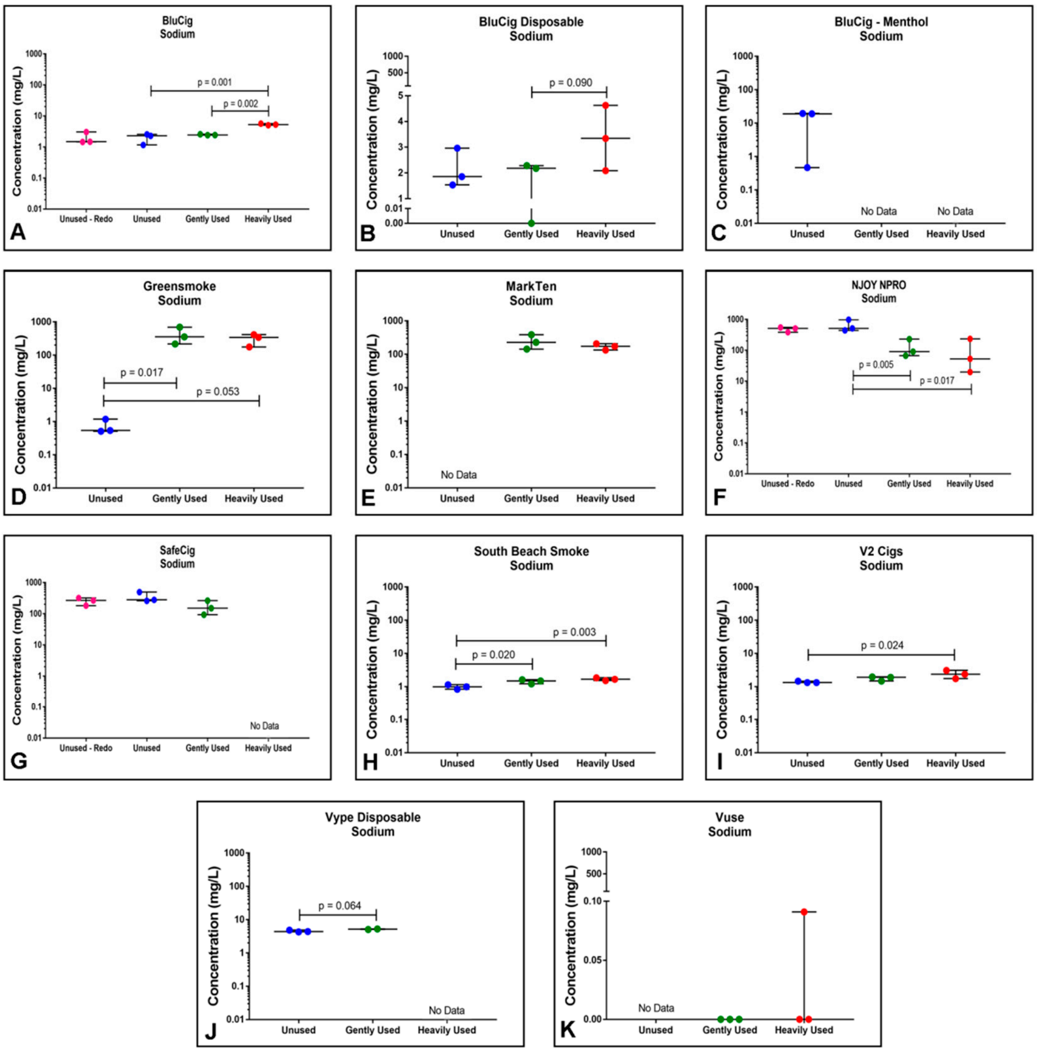

Figure S7. Sodium concentrations in ten brands of unused, gently used, and heavily used first generation EC fluid. (A) BluCig, (B) BluCig Disposable, (C) BluCig – Menthol, (D) Greensmoke, (E) MarkTen, (F) NJOY NPRO, (G) SafeCig, (H) South Beach Smoke, (I) V2 Cigs, (J) Vuse, and (K) Vype Disposable. All fluids were analyzed in triplicates. Blue = unused, green = gently used, red = heavily used, pink = retesting of unused fluid.

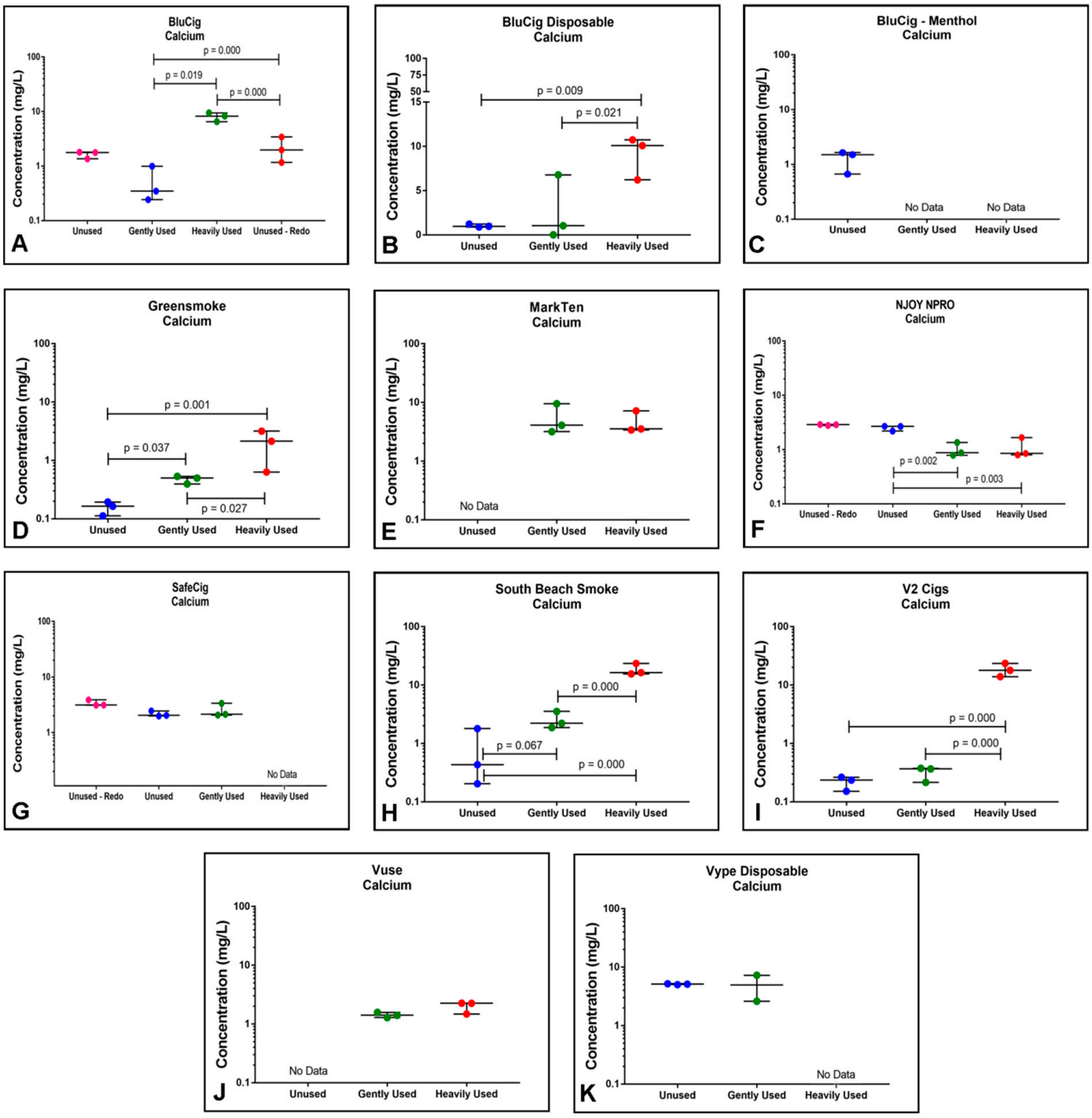

Figure S8. Calcium concentrations in ten brands of unused, gently used, and heavily used first generation EC fluid. (A) BluCig, (B) BluCig Disposable, (C) BluCig – Menthol, (D) Greensmoke, (E) MarkTen, (F) NJOY NPRO, (G) SafeCig, (H) South Beach Smoke, (I) V2 Cigs, (J) Vuse, and (K) Vype Disposable. All fluids were analyzed in triplicates. Blue = unused, green = gently used, red = heavily used, pink = retesting of unused fluid.

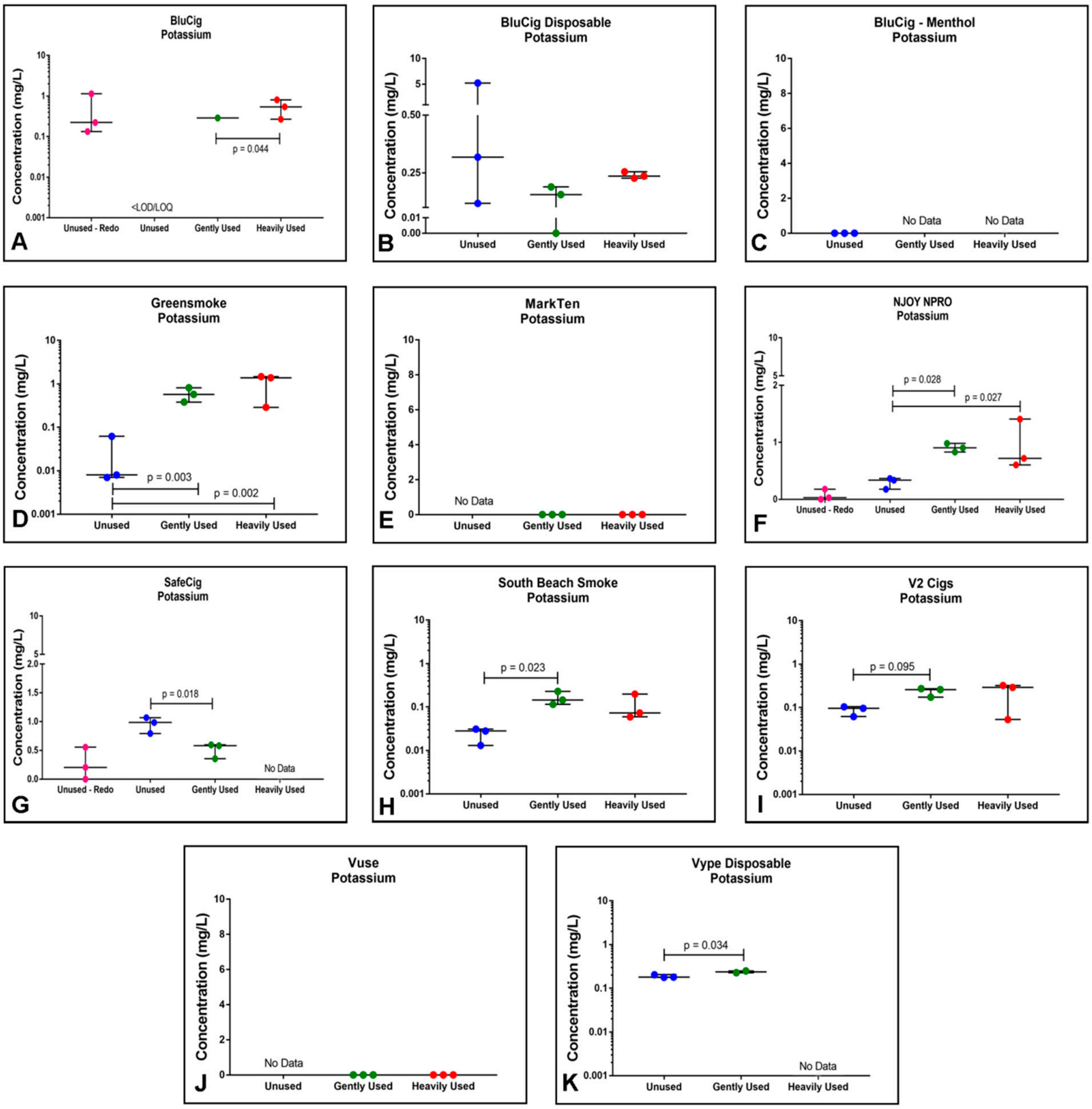

Figure S9. Potassium concentrations in ten brands of unused, gently used, and heavily used first generation EC fluid. (A) BluCig, (B) BluCig Disposable, (C) BluCig – Menthol, (D) Greensmoke, (E) MarkTen, (F) NJOY NPRO, (G) SafeCig, (H) South Beach Smoke, (I) V2 Cigs, (J) Vuse, and (K) Vype Disposable. All fluids were analyzed in triplicates. Blue = unused, green = gently used, red = heavily used, pink = retesting of unused fluid.

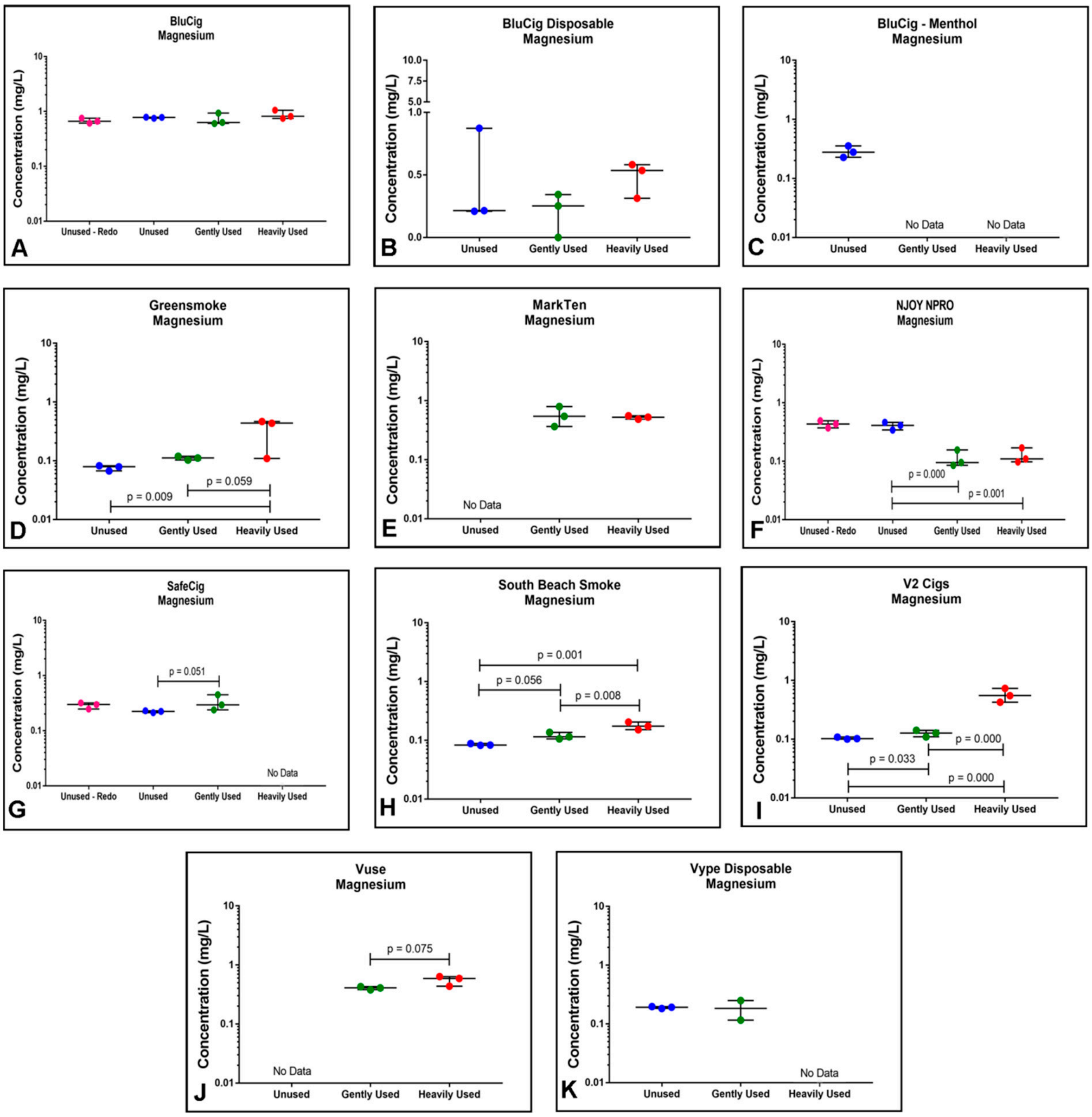

Figure S10. Magnesium concentrations in ten brands of unused, gently used, and heavily used first generation EC fluid. (A) BluCig, (B) BluCig Disposable, (C) BluCig – Menthol, (D) Greensmoke, (E) MarkTen, (F) NJOY NPRO, (G) SafeCig, (H) South Beach Smoke, (I) V2 Cigs, (J) Vuse, and (K) Vype Disposable. All fluids were analyzed in triplicates. Blue = unused, green = gently used, red = heavily used, pink = retesting of unused fluid.

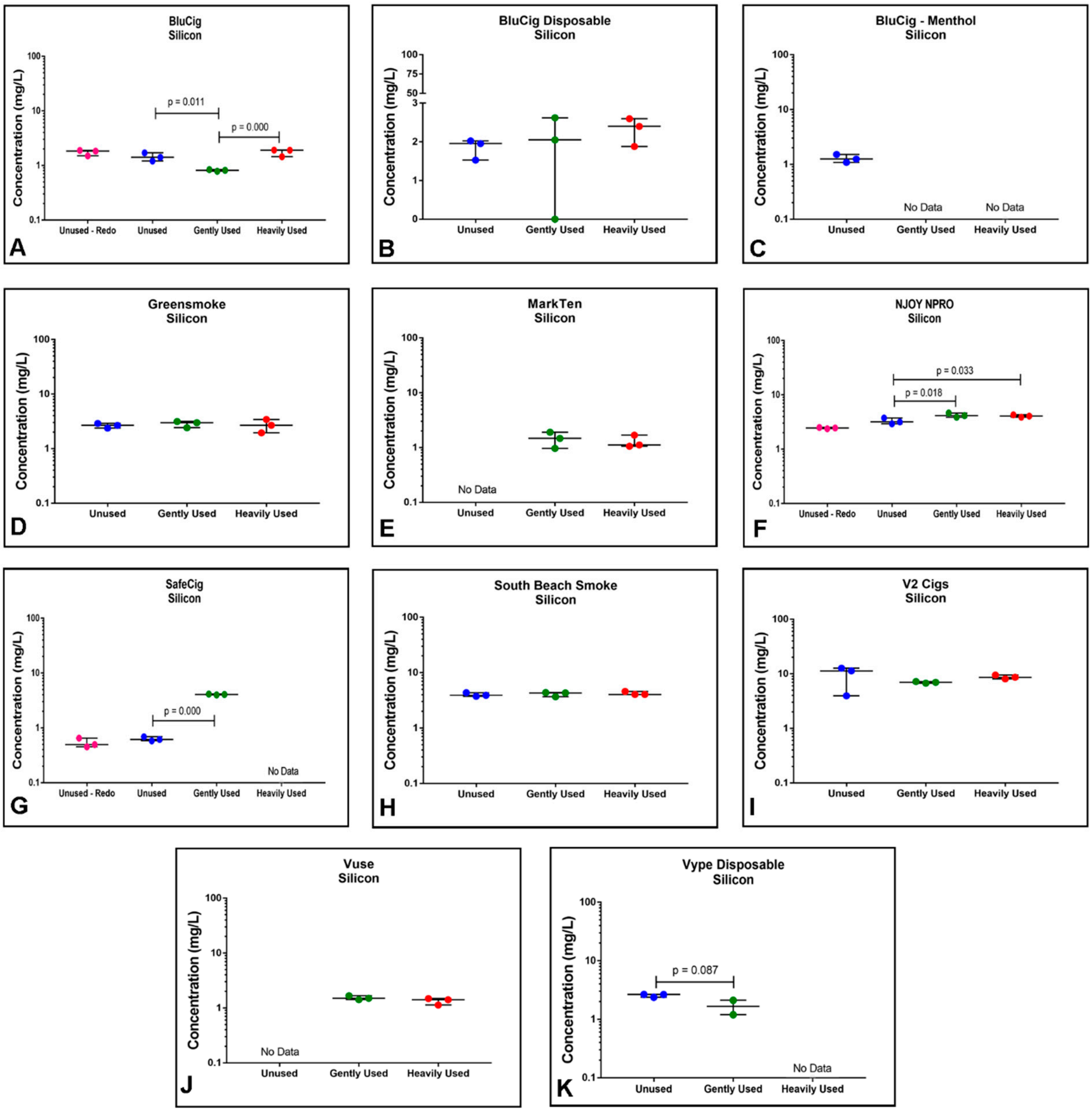

Figure S11. **Silicon concentrations in ten brands of unused, gently used, and heavily used first generation EC fluid.** (A) BluCig, (B) BluCig Disposable, (C) BluCig – Menthol, (D) Greensmoke, (E) MarkTen, (F) NJOY NPRO, (G) SafeCig, (H) South Beach Smoke, (I) V2 Cigs, (J) Vuse, and (K) Vype Disposable. All fluids were analyzed in triplicates. Blue = unused, green = gently used, red = heavily used, pink = retesting of unused fluid.

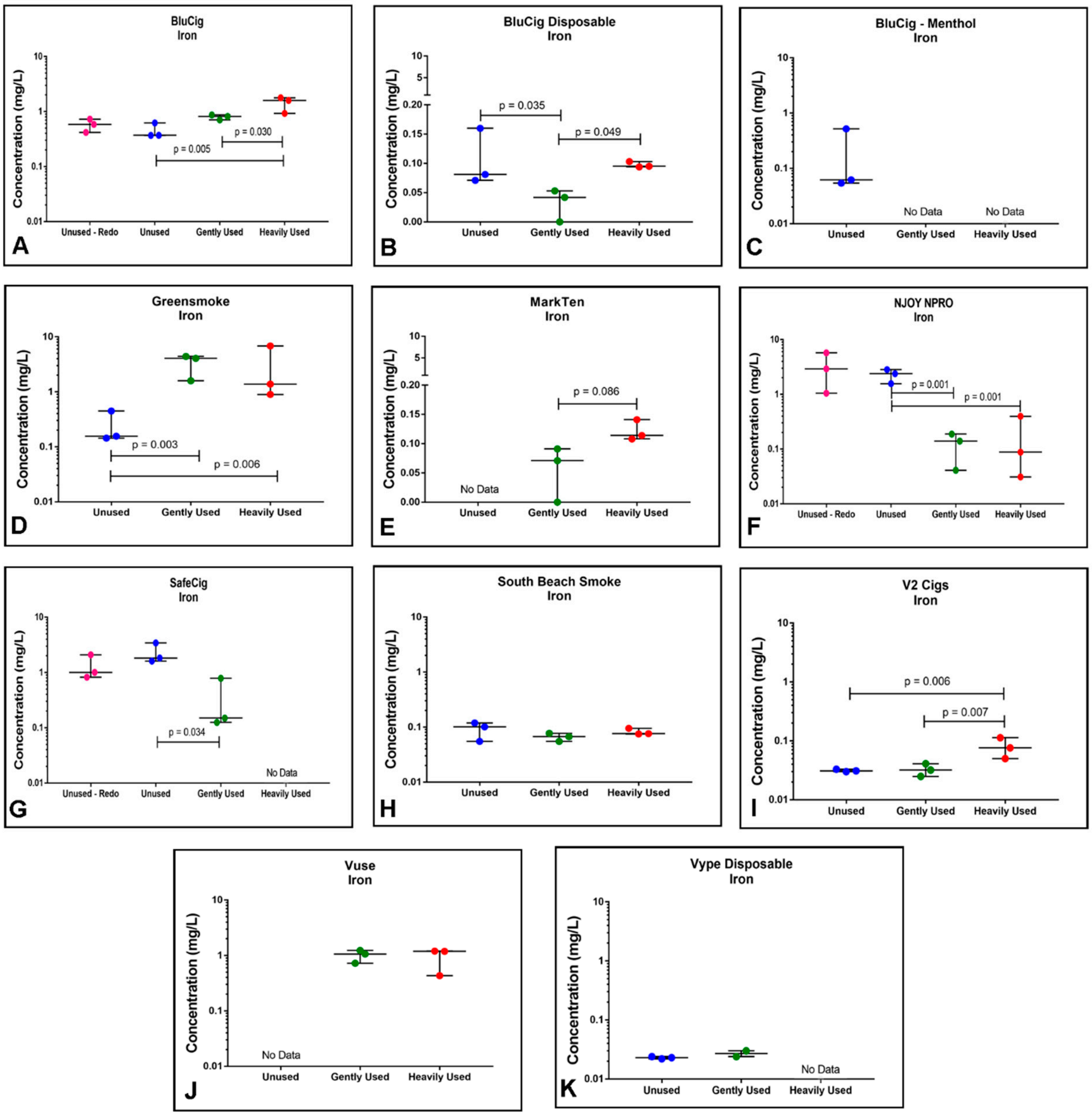

Figure S12. Iron concentrations in ten brands of unused, gently used, and heavily used first generation EC fluid. (A) BluCig, (B) BluCig Disposable, (C) BluCig – Menthol, (D) Greensmoke, (E) MarkTen, (F) NJOY NPRO, (G) SafeCig, (H) South Beach Smoke, (I) V2 Cigs, (J) Vuse, and (K) Vype Disposable. All fluids were analyzed in triplicates. Blue = unused, green = gently used, red = heavily used, pink = retesting of unused fluid.

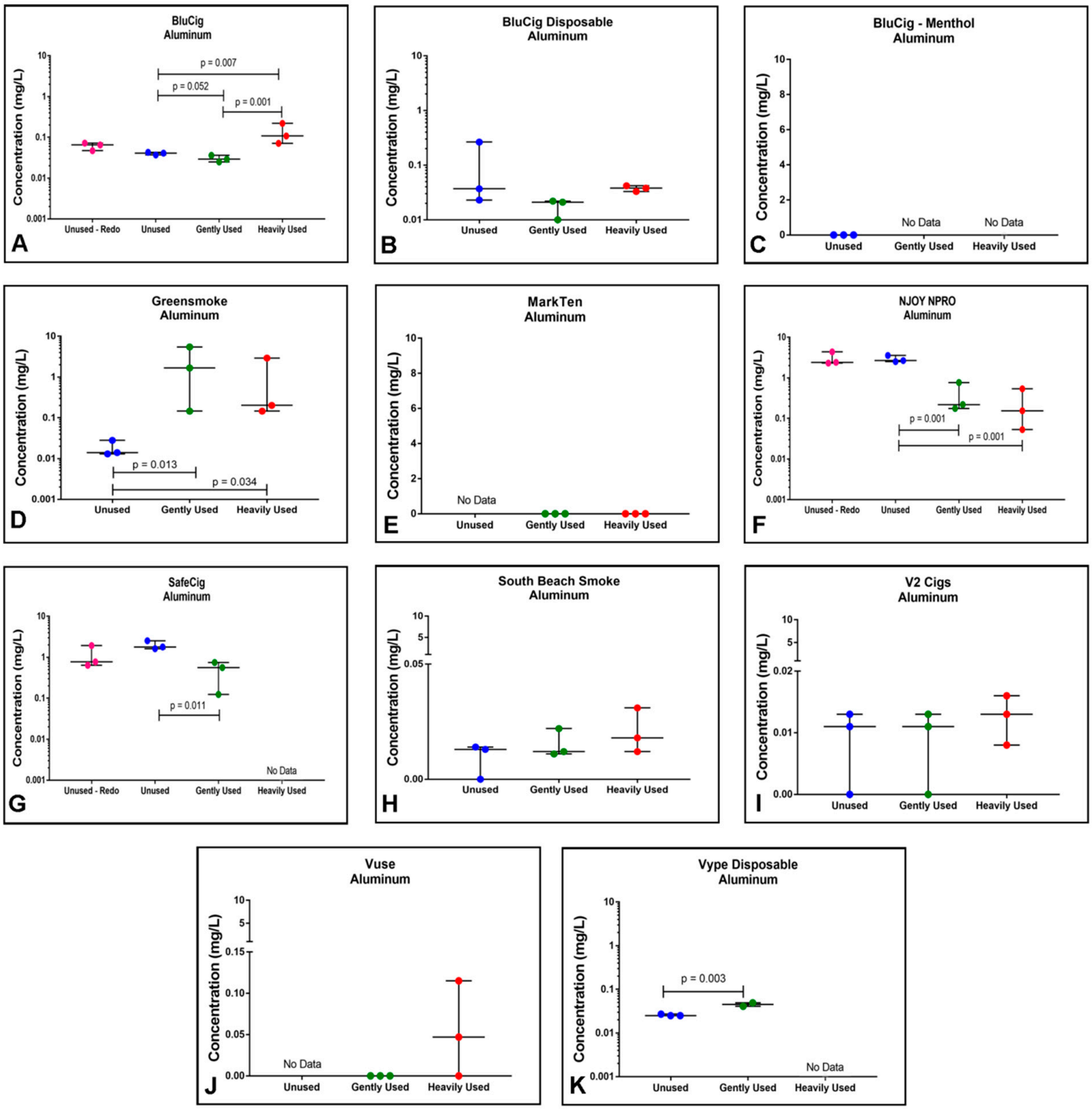

Figure S13. Aluminum concentrations in ten brands of unused, gently used, and heavily used first generation EC fluid. (A) BluCig, (B) BluCig Disposable, (C) BluCig – Menthol, (D) Greensmoke, (E) MarkTen, (F) NJOY NPRO, (G) SafeCig, (H) South Beach Smoke, (I) V2 Cigs, (J) Vuse, and (K) Vype Disposable. All fluids were analyzed in triplicates. Blue = unused, green = gently used, red = heavily used, pink = retesting of unused fluid.

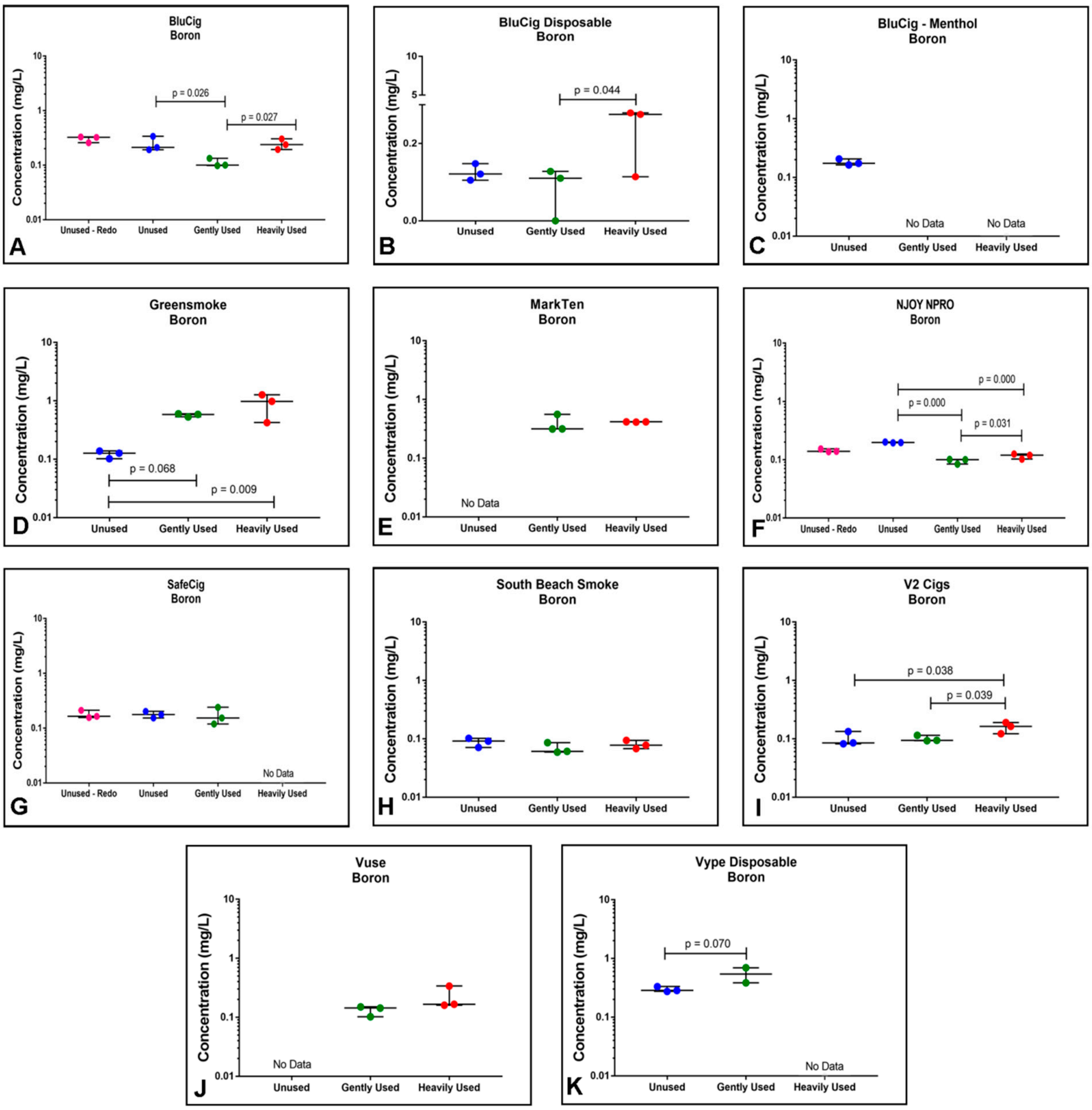

Figure S14. Boron concentrations in ten brands of unused, gently used, and heavily used first generation EC fluid. (A) BluCig, (B) BluCig Disposable, (C) BluCig – Menthol, (D) Greensmoke, (E) MarkTen, (F) NJOY NPRO, (G) SafeCig, (H) South Beach Smoke, (I) V2 Cigs, (J) Vuse, and (K) Vype Disposable. All fluids were analyzed in triplicates. Blue = unused, green = gently used, red = heavily used, pink = retesting of unused fluid.

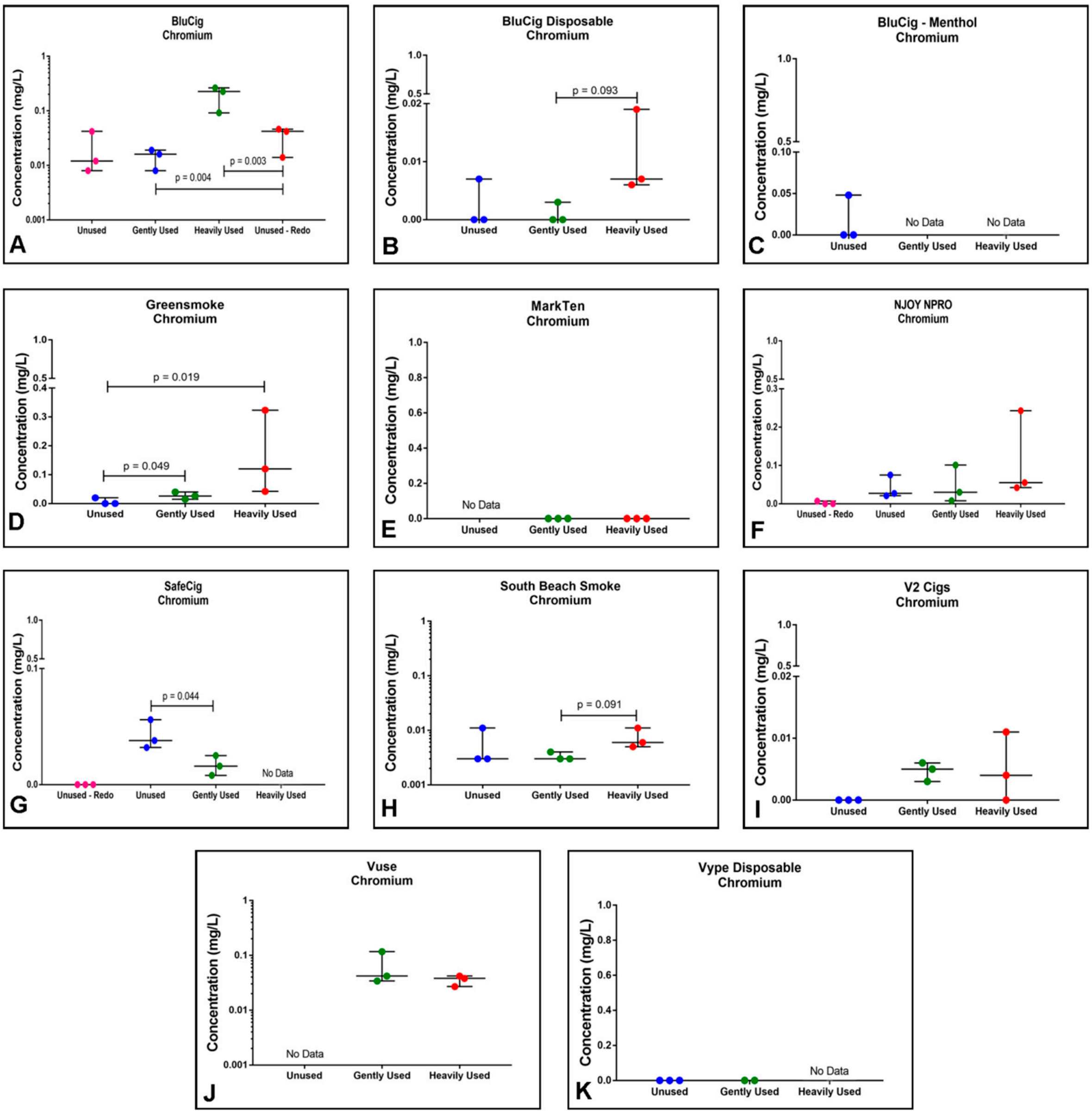

Figure S15. Chromium concentrations in ten brands of unused, gently used, and heavily used first generation EC fluid. (A) BluCig, (B) BluCig Disposable, (C) BluCig – Menthol, (D) Greensmoke, (E) MarkTen, (F) NJOY NPRO, (G) SafeCig, (H) South Beach Smoke, (I) V2 Cigs, (J) Vuse, and (K) Vype Disposable. All fluids were analyzed in triplicates. Blue = unused, green = gently used, red = heavily used, pink = retesting of unused fluid.

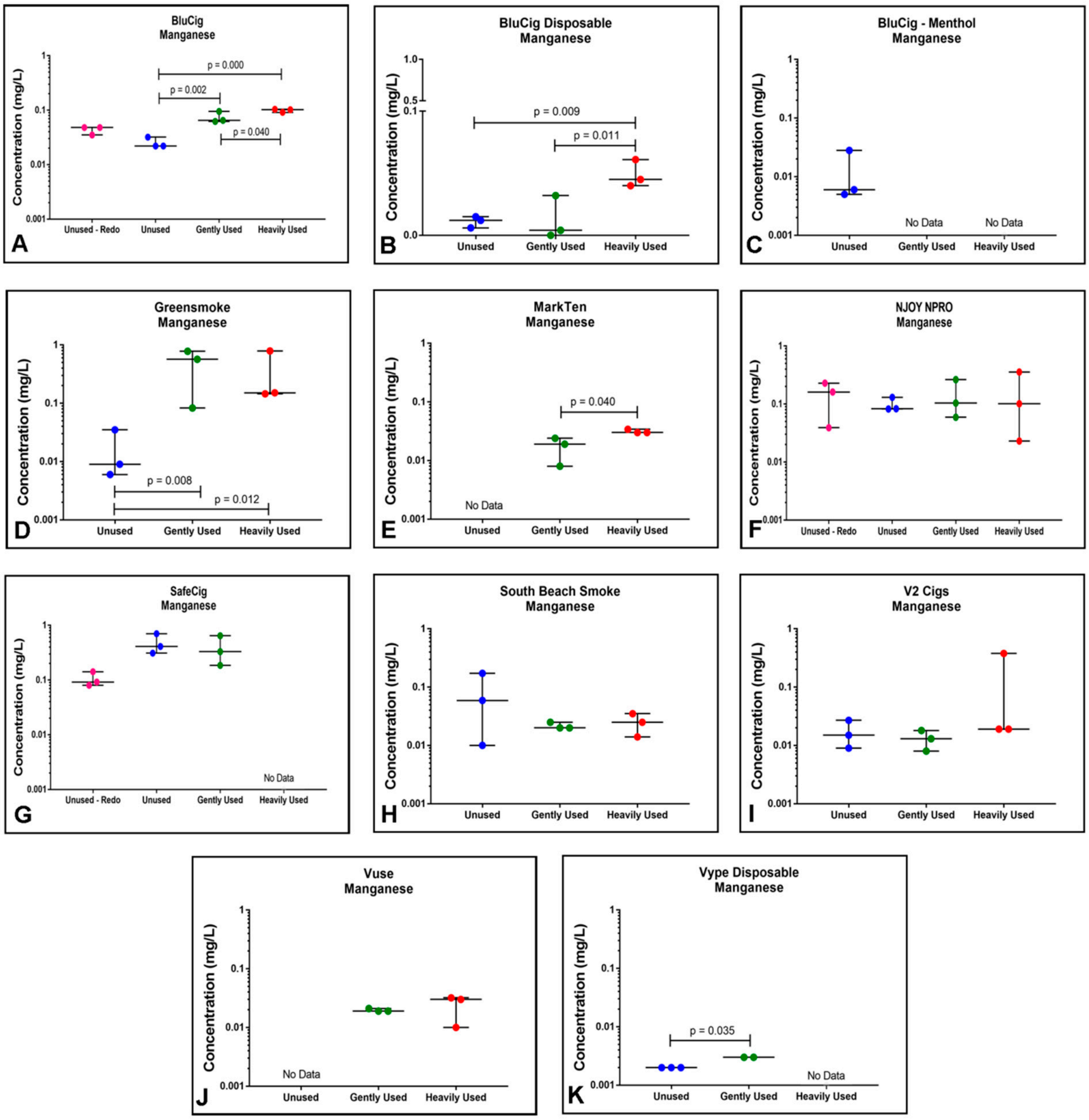

Figure S16. Manganese concentrations in ten brands of unused, gently used, and heavily used first generation EC fluid. (A) BluCig, (B) BluCig Disposable, (C) BluCig – Menthol, (D) Greensmoke, (E) MarkTen, (F) NJOY NPRO, (G) SafeCig, (H) South Beach Smoke, (I) V2 Cigs, (J) Vuse, and (K) Vype Disposable. All fluids were analyzed in triplicates. Blue = unused, green = gently used, red = heavily used, pink = retesting of unused fluid.

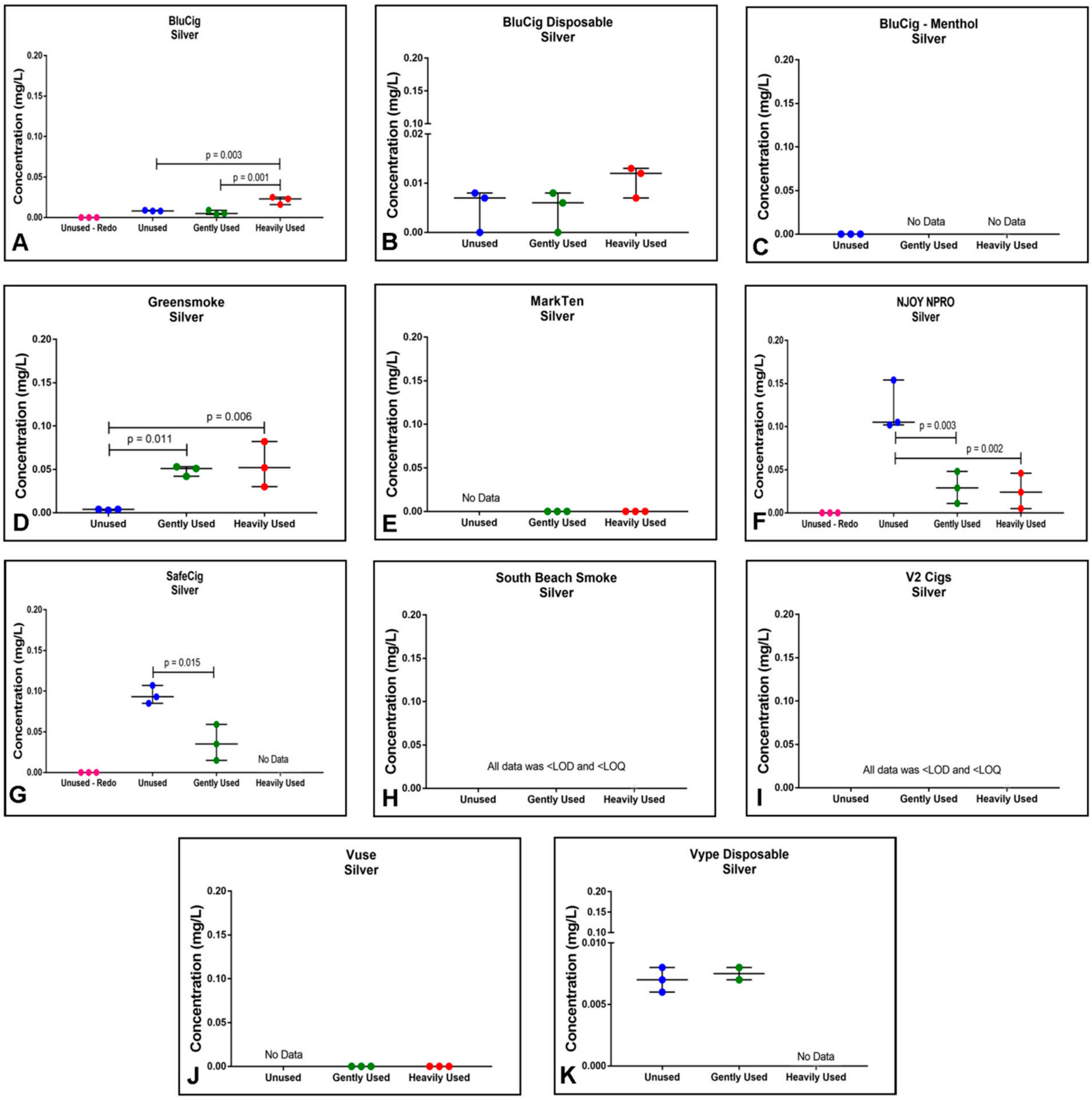

Figure S17. Silver concentrations in ten brands of unused, gently used, and heavily used first generation EC fluid. (A) BluCig, (B) BluCig Disposable, (C) BluCig – Menthol, (D) Greensmoke, (E) MarkTen, (F) NJOY NPRO, (G) SafeCig, (H) South Beach Smoke, (I) V2 Cigs, (J) Vuse, and (K) Vype Disposable. All fluids were analyzed in triplicates. Blue = unused, green = gently used, red = heavily used, pink = retesting of unused fluid.

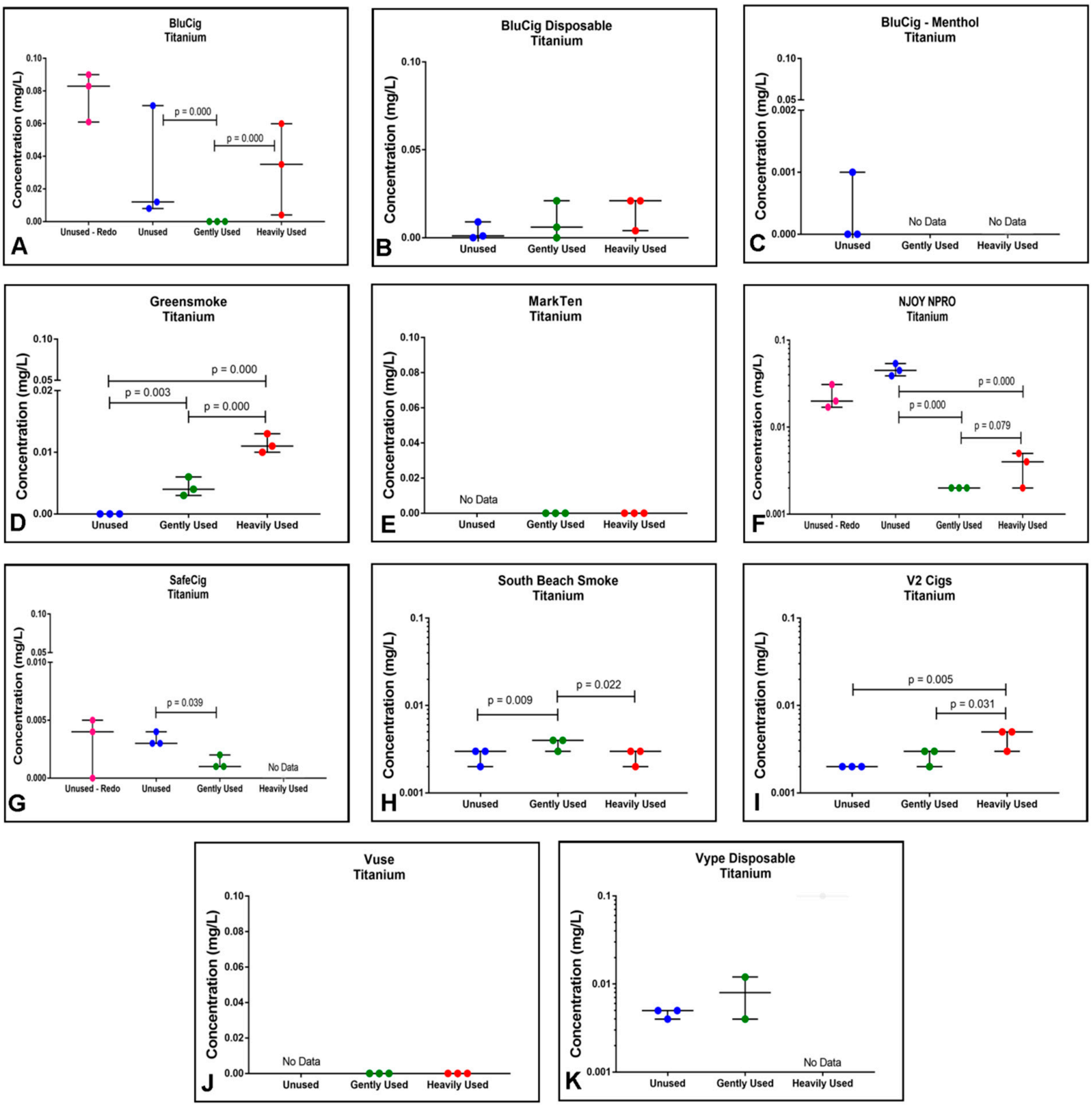

Figure S18. Titanium concentrations in ten brands of unused, gently used, and heavily used first generation EC fluid. (A) BluCig, (B) BluCig Disposable, (C) BluCig – Menthol, (D) Greensmoke, (E) MarkTen, (F) NJOY NPRO, (G) SafeCig, (H) South Beach Smoke, (I) V2 Cigs, (J) Vuse, and (K) Vype Disposable. All fluids were analyzed in triplicates. Blue = unused, green = gently used, red = heavily used, pink = retesting of unused fluid.

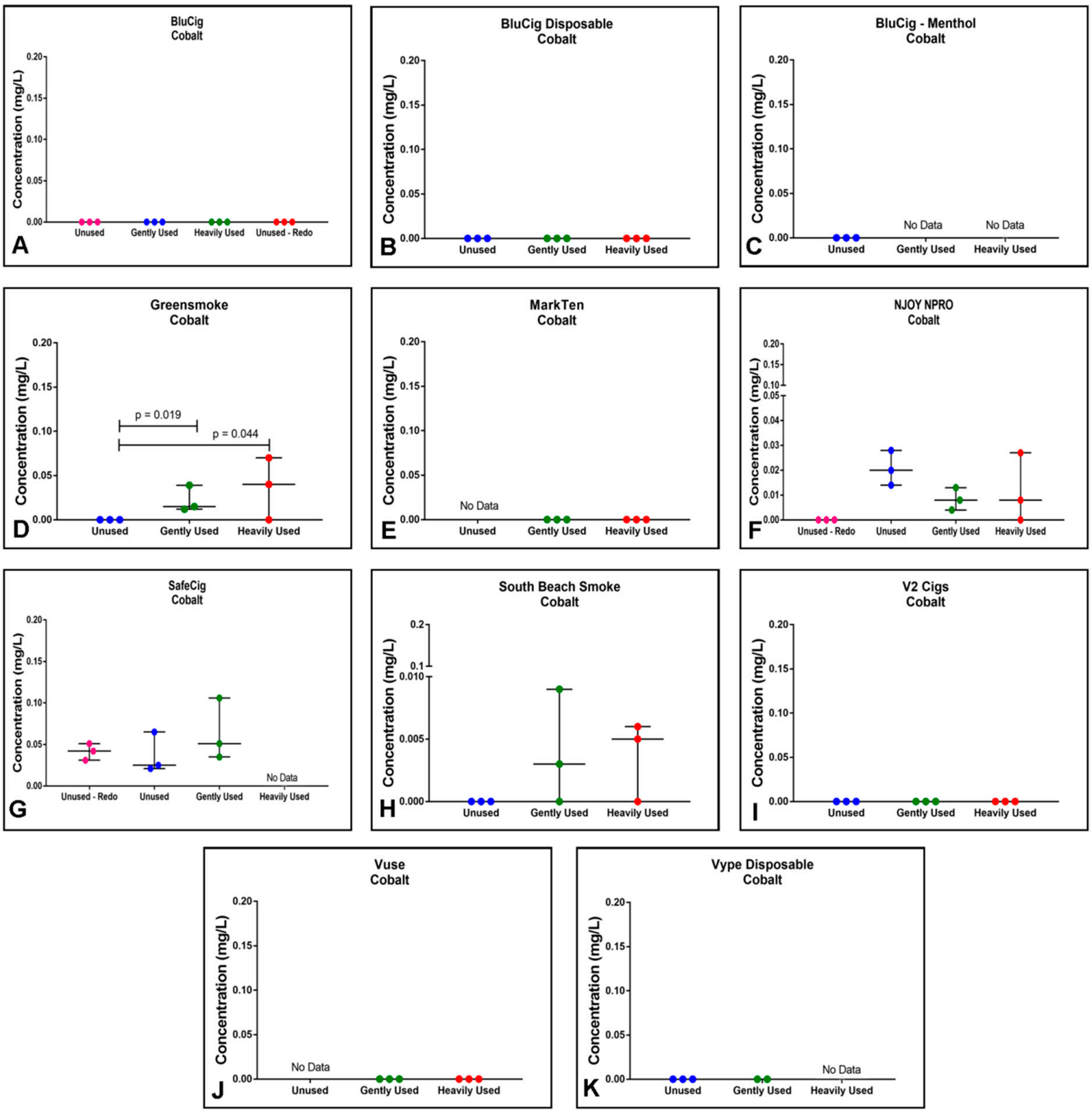

Figure S19. Cobalt concentrations in ten brands of unused, gently used, and heavily used first generation EC fluid. (A) BluCig, (B) BluCig Disposable, (C) BluCig – Menthol, (D) Greensmoke, (E) MarkTen, (F) NJOY NPRO, (G) SafeCig, (H) South Beach Smoke, (I) V2 Cigs, (J) Vuse, and (K) Vype Disposable. All fluids were analyzed in triplicates. Blue = unused, green = gently used, red = heavily used, pink = retesting of unused fluid.

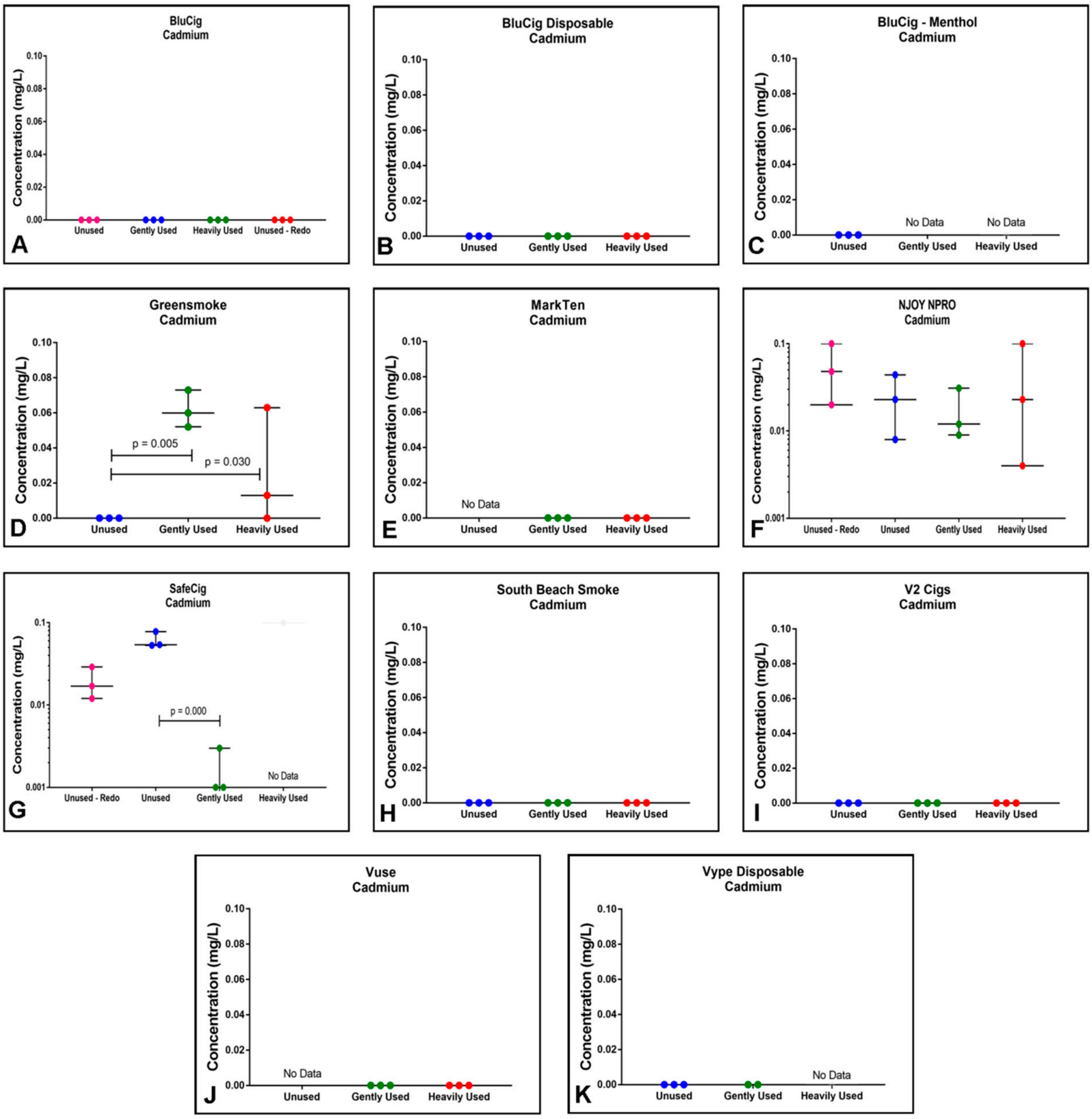

Figure S20. Cadmium concentrations in ten brands of unused, gently used, and heavily used first generation EC fluid. (A) BluCig, (B) BluCig Disposable, (C) BluCig – Menthol, (D) Greensmoke, (E) MarkTen, (F) NJOY NPRO, (G) SafeCig, (H) South Beach Smoke, (I) V2 Cigs, (J) Vuse, and (K) Vype Disposable. All fluids were analyzed in triplicates. Blue = unused, green = gently used, red = heavily used, pink = retesting of unused fluid.

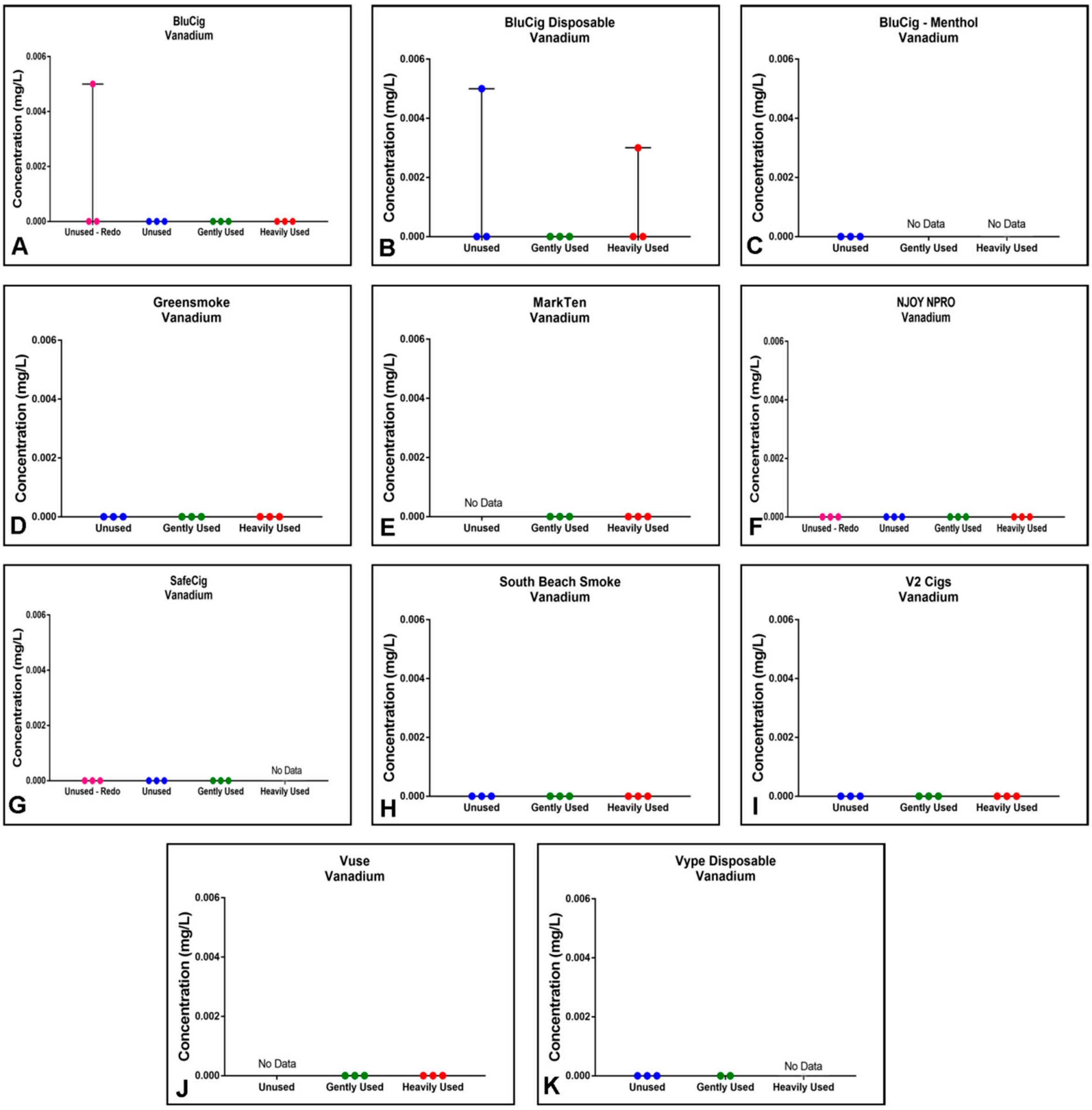

Figure S21. Vanadium concentrations in ten brands of unused, gently used, and heavily used first generation EC fluid. (A) BluCig, (B) BluCig Disposable, (C) BluCig – Menthol, (D) Greensmoke, (E) MarkTen, (F) NJOY NPRO, (G) SafeCig, (H) South Beach Smoke, (I) V2 Cigs, (J) Vuse, and (K) Vype Disposable. All fluids were analyzed in triplicates. Blue = unused, green = gently used, red = heavily used, pink = retesting of unused fluid.

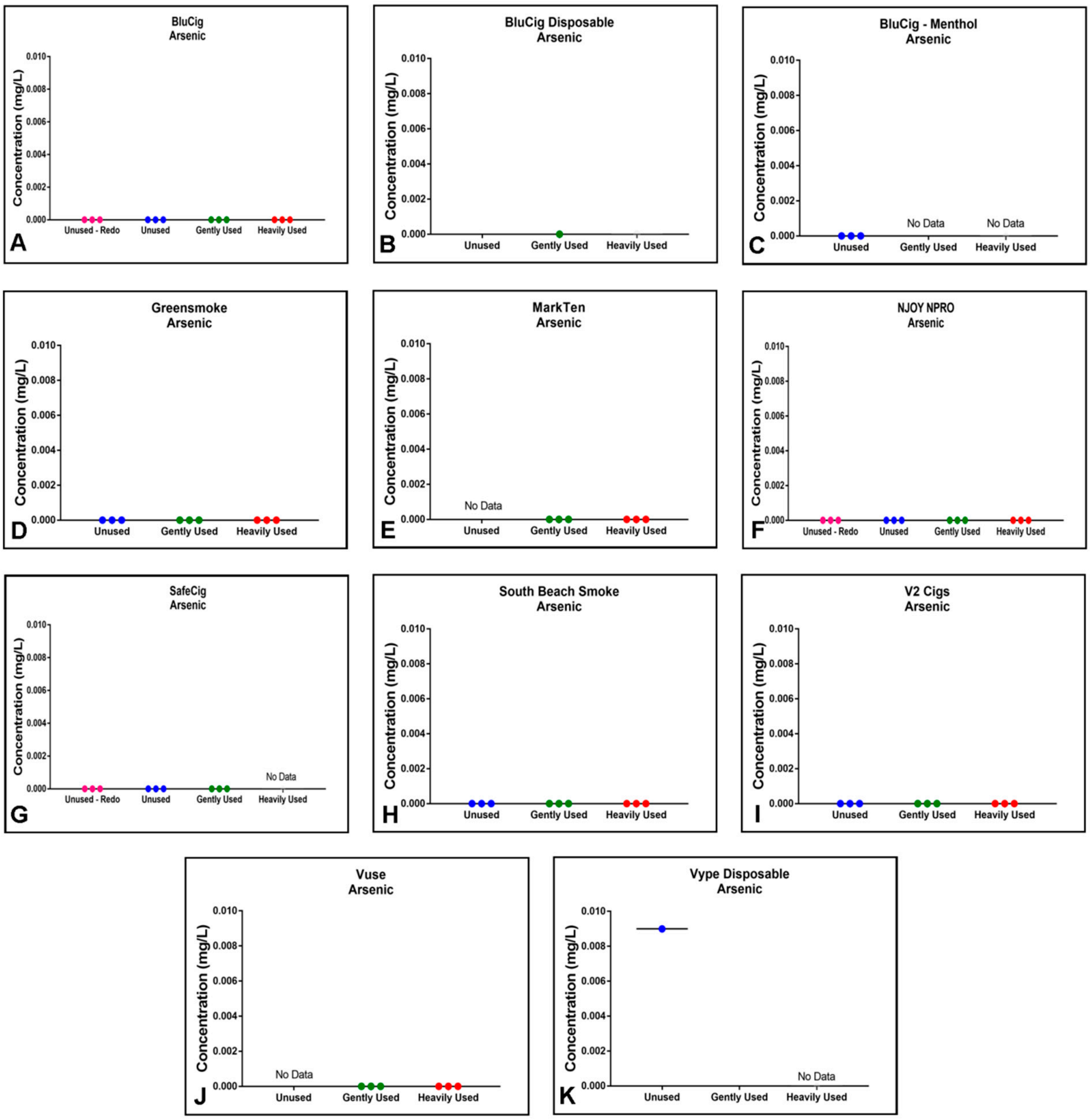

Figure S22. Arsenic concentrations in ten brands of unused, gently used, and heavily used first generation EC fluid. (A) BluCig, (B) BluCig Disposable, (C) BluCig – Menthol, (D) Greensmoke, (E) MarkTen, (F) NJOY NPRO, (G) SafeCig, (H) South Beach Smoke, (I) V2 Cigs, (J) Vuse, and (K) Vype Disposable. All fluids were analyzed in triplicates. Blue = unused, green = gently used, red = heavily used, pink = retesting of unused fluid.

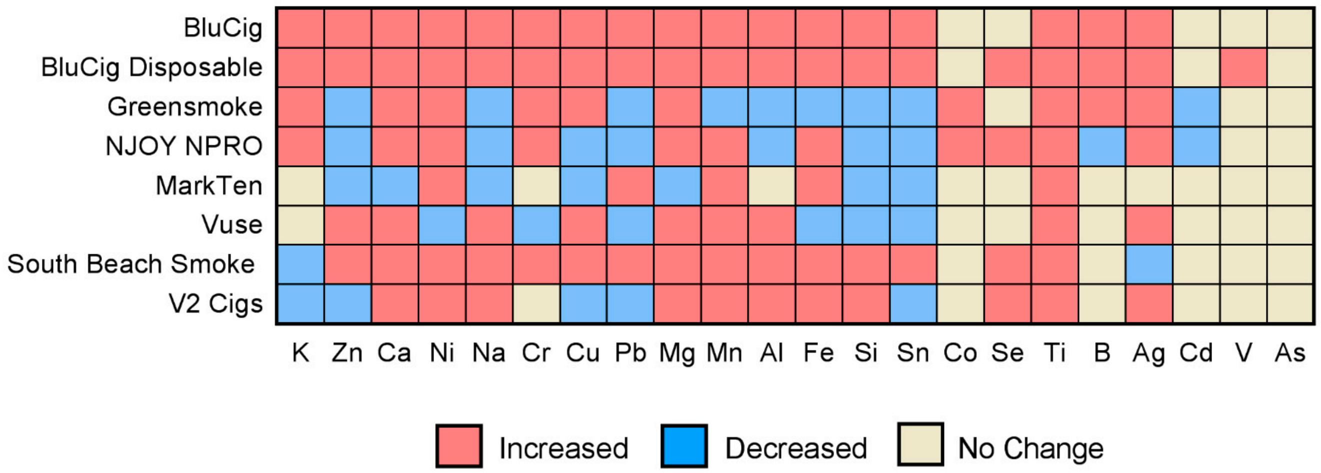

Figure S23. Heat map showing element concentrations in heavily used EC fluids relative to gently used ECs fluids. In 8 EC brands, a majority of the 22 element concentrations were higher in heavily used EC fluids when compared to gently used fluids. Red squares = increase, blue squares = decrease, tan squares = no change.

Table S8: Relationship between elements detected in unused (U), gently used (G), and heavily used (H) e-liquids and elements identified in EC atomizer components (Y).

Element	Brand	Detected in Fluid	Identified in EC Components						
			Filament	Thick Wire	Wire-Wire Joint	Wire-Air Tube Joint	Air Tube	Wick	Sheath
Aluminum	BluCig	U, G, H						Y	Y
	BluCig Disposable	U, G, H						Y	Y
	Greensmoke	U, G, H						Y	
	Mark Ten							Y	Y
	NJOY NPRO	U, G, H							Y
	SafeCig	U, G							Y
	South Beach Smoke	U, G, H							Y
	V2 Cigs	U, G, H						Y	Y
	Vuse	H							
	Vype	U, G							Y
Calcium	BluCig	U, G, H							Y
	BluCig Disposable	U, G, H						Y	Y
	Greensmoke	U, G, H				Y		Y	Y
	Mark Ten	G, H							Y
	NJOY NPRO	U, G, H							Y
	SafeCig	U, G							Y
	South Beach Smoke	U, G, H							Y
	V2 Cigs	U, G, H						Y	Y
	Vuse	G, H						Y	
	Vype	U, G							Y
Chromium	BluCig	U, G, H	Y		Y				
	BluCig Disposable	U, G, H	Y						
	Greensmoke	U, G, H	Y						
	Mark Ten		Y						
	NJOY NPRO	U, G, H	Y						
	SafeCig	G, H	Y						
	South Beach Smoke	U, G, H	Y						
	V2 Cigs	G, H	Y		Y				
	Vuse	G, H	Y						
	Vype		Y						

Cobalt	BluCig								
	BluCig Disposable								
	Greensmoke	G, H							
	Mark Ten								
	NJOY NPRO	U, G, H							
	SafeCig	U, G							
	South Beach Smoke	G, H					Y		
	V2 Cigs								
	Vuse								
	Vype								
Copper	BluCig	U, G, H	Y	Y	Y		Y		
	BluCig Disposable	U, G, H		Y					
	Greensmoke	U, G, H		Y	Y		Y		
	Mark Ten	G, H		Y	Y	Y			
	NJOY NPRO	U, G, H		Y	Y		Y		
	SafeCig	U, G	Y	Y	Y	Y			
	South Beach Smoke	U, G, H					Y		
	V2 Cigs	U, G, H				Y	Y		
	Vuse	G, H	Y						
	Vype	U, G		Y	Y				
Iron	BluCig	U, G, H			Y		Y		
	BluCig Disposable	U, G, H							
	Greensmoke	U, G, H							
	Mark Ten	G, H							
	NJOY NPRO	U, G, H							
	SafeCig	U, G	Y						
	South Beach Smoke	U, G, H	Y				Y		
	V2 Cigs	U, G, H	Y						
	Vuse	G, H	Y						
	Vype	U, G							
Lead	BluCig	U, G, H				Y	Y		
	BluCig Disposable	U, G, H							
	Greensmoke	G, H							

	Mark Ten	G, H							
	NJOY NPRO	U, G, H							
	SafeCig	U, G							
	South Beach Smoke	U, G, H					Y		
	V2 Cigs	G, H				Y			
	Vuse	G, H							
	Vype	U, G							
Magnesium	BluCig	U, G, H						Y	Y
	BluCig Disposable	U, G, H						Y	Y
	Greensmoke	U, G, H						Y	Y
	Mark Ten	G, H							Y
	NJOY NPRO	U, G, H							Y
	SafeCig	U, G							
	South Beach Smoke	U, G, H							Y
	V2 Cigs	U, G, H							Y
	Vuse	G, H						Y	
	Vype	U, G							Y
Manganese	BluCig	U, G, H							
	BluCig Disposable	U, G, H							
	Greensmoke	U, G, H							
	Mark Ten	G, H							
	NJOY NPRO	U, G, H							
	SafeCig	U, G							
	South Beach Smoke	U, G, H	Y						
	V2 Cigs	U, G, H							
	Vuse	G, H							
	Vype	U, G							
Nickel	BluCig	U, G, H	Y		Y		Y		
	BluCig Disposable	U, G, H	Y						
	Greensmoke	U, G, H	Y				Y		
	Mark Ten	G, H	Y				Y		
	NJOY NPRO	U, G, H	Y				Y		
	SafeCig	U, G	Y				Y		
	South Beach Smoke	U, G, H	Y				Y		

	V2 Cigs	U, G, H	Y	Y	Y		Y		
	Vuse	G, H	Y						
	Vype	U, G	Y						
Potassium	BluCig	U, G, H							Y
	BluCig Disposable	U, G, H							
	Greensmoke	U, G, H							
	Mark Ten								
	NJOY NPRO	U, G, H							Y
	SafeCig	U, G							
	South Beach Smoke	U, G, H							
	V2 Cigs	U, G, H							
	Vuse							Y	
	Vype	U, G							
Silicon	BluCig	U, G, H						Y	Y
	BluCig Disposable	U, G, H						Y	Y
	Greensmoke	U, G, H						Y	Y
	Mark Ten	G, H						Y	Y
	NJOY NPRO	U, G, H						Y	Y
	SafeCig	U, G						Y	Y
	South Beach Smoke	U, G, H						Y	Y
	V2 Cigs	U, G, H						Y	Y
	Vuse	G, H						Y	
	Vype	U, G						Y	Y
Silver	BluCig	U, G, H		Y					
	BluCig Disposable	U, G, H		Y					
	Greensmoke	U, G, H		Y					
	Mark Ten			Y			Y		
	NJOY NPRO	U, G, H		Y					
	SafeCig	U, G		Y			Y		
	South Beach Smoke								
	V2 Cigs						Y		
	Vuse		Y						
	Vype	U, G		Y					

Sodium	BluCig	U, G, H							Y
	BluCig Disposable	U, G, H							
	Greensmoke	U, G, H							
	Mark Ten	G, H							
	NJOY NPRO	U, G, H							Y
	SafeCig	U, G							
	South Beach Smoke	U, G, H							
	V2 Cigs	U, G, H							
	Vuse	H						Y	
	Vype	U, G							
Tin	BluCig	U, G, H				Y			
	BluCig Disposable	U, G, H				Y			
	Greensmoke	U, G, H				Y			
	Mark Ten	G, H				Y			
	NJOY NPRO	U, G, H		Y					
	SafeCig	U, G				Y			
	South Beach Smoke	U, G, H					Y		
	V2 Cigs	U, G, H							
	Vuse	G, H							
	Vype	U, G				Y			
Zinc	BluCig	U, G, H					Y		
	BluCig Disposable	U, G, H			Y				
	Greensmoke	U, G, H			Y		Y		
	Mark Ten	G, H			Y				
	NJOY NPRO	U, G, H					Y		
	SafeCig	U, G			Y	Y	Y		
	South Beach Smoke	U, G, H					Y		
	V2 Cigs	U, G, H				Y	Y		
	Vuse	G, H							
	Vype	U, G			Y				

Table S9. Flavor chemicals and acids in aged unused and used e-liquids that were below limit of quantification or not detected

**Flavor Chemicals Detected <LOQ**		**Acids – Not Detected**
Furfural	Hemineurine	Benzoic acid
Furfuryl alcohol	Eugenol	Salicylic acid
2,5-dimethylpyrazine	Piperonal	Propionic acid
2,3,5-Trimethylpyrazine	α-Damascone	Maleic acid
Limonene	β-Damascone	
Acetylpyrazine	Hydrocoumarin	
Benzyl Alcohol	Ethyl Vanillin	
2,3,5,6-Tetramethylpyrazine	δ-Decalactone	
Phenethyl alcohol	Raspberry ketone	
Menthol	Gingerone	
α-Terpineol		

Table S10. Concentrations of elements/metals reported in the e-liquids (µg/mg)

Means	Flora et al 2016	Hess et al 2017	Dunbar et al 2018	Zhao et al 2018	Olmedo et al 2018	Williams et al 2020		Current Study
EC type	Cartridge	Cartridge	Cartridge	Bottle/Cartridge	Tank	Cartomizer	Tank	Cartridge
Aluminum					101	15.5	6.03-575	6.9-2091
Arsenic	<430				4.2	15.5-56.0	36.2-79.3	
Cadmium	<220	0.4-176			0.4			1.72-53.4
Cobalt					10.8			3.45-55.2
Chromium		46.4 - 1815			214	0.86	0.86-1673.3	0.86-167.2
Copper					1990	115.5-3278.4	44.0-1050.9	134.5-366043.1
Iron					1880	17.2-282.8	0.86-78726.7	23.3-2892.2
Manganese		24.7-5943			124			2.59-411.2
Nickel		50.5-19436			2510	10.3	56.0-4325	0.86-61287.1
Lead		4.2-1694	<7.8		517			12.1-40312.9
Zinc				220	3250	121.6-2058.6	360.3-1644.8	440.5-254991.4

Color code: grey = higher in current study.

Table S11. Comparison of element/metal concentrations (mg/L) in cigarette butts (CB) and EC cartomizers

	Moerman, Potts 2011	Williams et al 2022	Moerman, Potts 2011	Williams et al 2022
CB = Day 34	Non-smoked CB	Unused	Smoked CB	Aged Used
Aluminum	6.9	4.831	8.92	5.46
Cadmium	0.132	0.078	0.142	0.073
Chromium	0.283	0.075	0.236	0.323
Copper	0.67	1749	1.22	608.2
Iron	75.3	57.28	45.3	6.82
Lead	1.79	93.38	1.42	72.9
Manganese	59.6	0.701	40.1	0.79
Nickel	0.404	78.29	0.298	102.49
Titanium	0.729	0.09	0.755	0.06
Zinc	9.68	528.67	5.87	435.48

Color code: red = higher overall in CBs, grey = similar in both, blue = higher overall in ECs, orange = higher in CB, green = higher in EC.
